# Polθ is phosphorylated by PLK1 to repair double-strand breaks in mitosis

**DOI:** 10.1038/s41586-023-06506-6

**Published:** 2023-09-06

**Authors:** Camille Gelot, Marton Tibor Kovacs, Simona Miron, Emilie Mylne, Alexis Haan, Liza Boeffard-Dosierre, Rania Ghouil, Tatiana Popova, Florent Dingli, Damarys Loew, Josée Guirouilh-Barbat, Elaine Del Nery, Sophie Zinn-Justin, Raphael Ceccaldi

**Affiliations:** 1 INSERM U830, PSL Research University, Institut Curie, Paris, France; 2 Institute for Integrative Biology of the Cell (I2BC), CEA, CNRS, Paris-Saclay University, Gif-sur-Yvette, France; 3 INSERM U830, DNA Repair and Uveal Melanoma (D.R.U.M.), Equipe labellisée par la Ligue Nationale Contre le Cancer, PSL Research University, Institut Curie, Paris, France; 4 CurieCoreTech Mass Spectrometry Proteomics, Institut Curie, PSL Research University, Paris, France; 5 Université de Paris, INSERM U1016, UMR 8104 CNRS, Equipe Labellisée Ligue Nationale Contre le Cancer, Institut Cochin, Paris, France; 6 Department of Translational Research-Biophenics High-Content Screening Laboratory, Cell and Tissue Imaging Facility (PICT-IBiSA), PSL Research University, Institut Curie, Paris, France

**Keywords:** Double-strand DNA breaks, DNA synthesis, Breast cancer

## Abstract

DNA double-strand breaks (DSBs) are deleterious lesions that challenge genome integrity. To mitigate this threat, human cells rely on the activity of multiple DNA repair machineries that are tightly regulated throughout the cell cycle^1^. In interphase, DSBs are mainly repaired by non-homologous end joining and homologous recombination^2^. However, these pathways are completely inhibited in mitosis^3–5^, leaving the fate of mitotic DSBs unknown. Here we show that DNA polymerase theta^6^ (Polθ) repairs mitotic DSBs and thereby maintains genome integrity. In contrast to other DSB repair factors, Polθ function is activated in mitosis upon phosphorylation by Polo-like kinase 1 (PLK1). Phosphorylated Polθ is recruited by a direct interaction with the BRCA1 C-terminal domains of TOPBP1 to mitotic DSBs, where it mediates joining of broken DNA ends. Loss of Polθ leads to defective repair of mitotic DSBs, resulting in a loss of genome integrity. This is further exacerbated in cells that are deficient in homologous recombination, where loss of mitotic DSB repair by Polθ results in cell death. Our results identify mitotic DSB repair as the underlying cause of synthetic lethality between Polθ and homologous recombination. Together, our findings reveal the critical importance of mitotic DSB repair in the maintenance of genome integrity.

## Abstract (web summary)

In mitosis, genome integrity is maintained by DNA polymerase theta-dependent repair of DNA double-strand breaks, which is regulated by Polo-like kinase 1 activity.

## Main

Cells can enter mitosis with DSBs that are formed during interphase^7^, and DSBs can also form in mitosis as a consequence of replication stress^8,9^. As a result, cells that are deficient in homologous recombination (HR), which experience increased levels of replication stress, are prone to accumulate mitotic DSBs^10^. It has been shown that broken DSB ends can be held together by a tethering complex and pass through mitosis in this manner, awaiting repair during the next cell cycle^11,12^. However, whether this pathway is sufficient to safeguard the genome against mitotic DSBs remains unclear. An intriguing possibility is that DSB repair pathways that are neither HR nor non-homologous end joining^13,14^ (NHEJ) might be active in mitosis^15–20^. DNA polymerase theta (Polθ) has recently emerged as having a major role in alternative end-joining repair^21–23^, which is essential for the survival of HR-deficient cells^24–27^. Here we reasoned that Polθ-mediated end joining could have a key role in repairing mitotic DSBs, thereby maintaining genome integrity and ensuring the survival of HR-deficient cells.

## HR-dependent Polθ recruitment in S phase

**Fig. 1:**
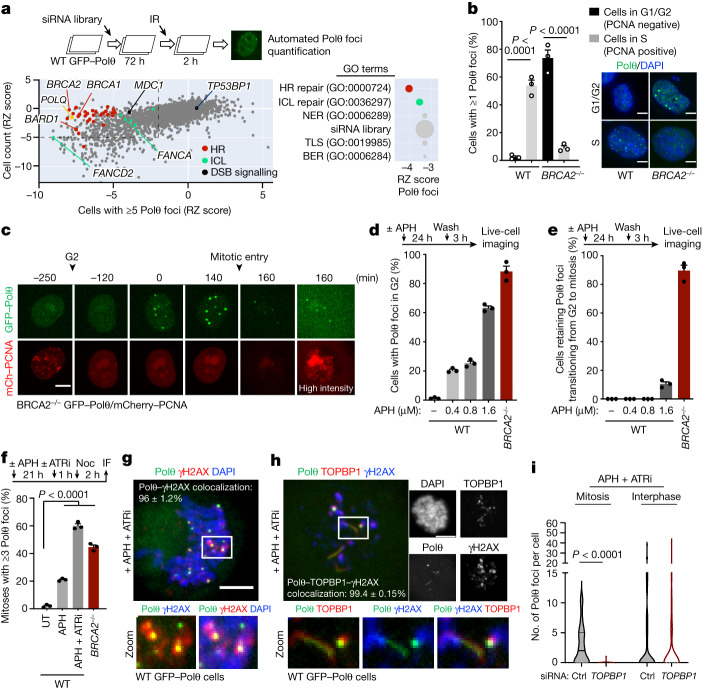
Polθ is differentially recruited to DSBs depending on HR status and cell cycle phase. **a**, Top, schematic of siRNA screen for ionizing radiation (IR)-induced Polθ foci formation. Bottom, robust *Z*-score (RZ score) for cell survival and Polθ foci formation for siRNA against indicated targets. For each siRNA, the median RZ score of three replicate experiments is shown. Arrows show the strongest RZ score for the indicated gene. Right, enriched Gene Ontology (GO) terms for biological processes identified among all targets. BER, base excision repair; ICL, interstrand crosslink repair; NER, nucleotide excision repair; TLS, translesion synthesis. **b**, Cell cycle distribution of Polθ foci in wild-type (WT) and *BRCA2*^*−/−*^ cells (left), as determined by confocal microscopy (right). WT G1/G2: *n* = 407; WT S: *n* = 137; *BRCA2*^*−/−*^ G1/G2: 119; *BRCA2*^*−/−*^ S: *n* = 187. **c**, Representative images from live microscopy analysis of *BRCA2*^*−/−*^ cells expressing GFP–Polθ and mCherry (mCh)–PCNA. **d**, Quantification of Polθ foci in G2 in wild-type and *BRCA2*^*−/−*^ cells with indicated doses of aphidicolin (APH). Top, schematic of the experiment. **e**, Quantification of cells retaining Polθ foci while transitioning from G2 to mitosis. **d**,**e**, At least 30 cells were analysed for each condition per experiment. **f**, Quantification of Polθ foci in mitotic cells upon indicated treatment (aphidicolin: 0.4 μM, 24 h). Untreated (UT): *n* = 165; aphidicolin: *n* = 87; aphidicolin + ATR inhibitor VE-821 (ATRi): *n* = 152; *BRCA2*^*−/−*^: *n* = 165. IF, immunofluorescence; Noc, nocodazole. **g**,**h**, Representative images and quantification of colocalization of Polθ foci (**g**) and filaments (**h**) with γH2AX and TOPBP1 in mitosis. **i**, Quantification of Polθ foci formation upon indicated treatment in interphase or mitotic cells. All scale bars represent 5 μm. Mitosis control (Ctrl): *n* = 58; mitosis *TOPBP1*: *n* = 38; interphase control: *n* = 53; interphase *TOPBP1*: *n* = 83. Data represent three biological replicates. Data are mean ± s.e.m, except in violin plots (**i**), which show median with quartiles. **b**,**f**, Chi-square test. **b**, Wild type, χ^2^ = 43.6427; *BRCA2*^*−/−*^, χ^2^ = 130.003. **f**, aphidicolin + ATRi, χ^2^ = 129.9292; *BRCA2*^*−/−*^, χ^2^ = 85.7293. **i**, Kruskal–Wallis test corrected with Dunn’s multiple comparisons test.

**Extended Data Fig. 1:**
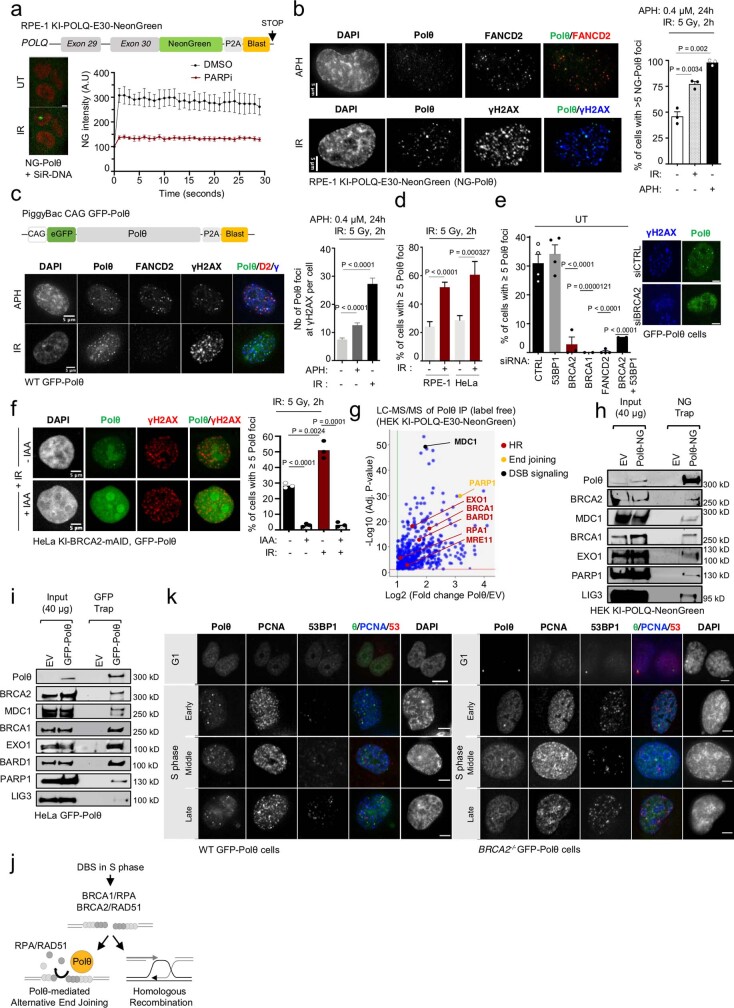
related to Fig. 1. Polθ foci formation in different cell cycle phases varies with HR status. **a**, Scheme representing endogenously tagged *POLQ* locus. Representative images and quantification of endogenous Neon Green (NG)-tagged Polθ recruitment to laser microirradiated loci in RPE-1 cells. **b**, Representative images and quantification of endogenous NG-Polθ foci formation upon indicated treatment. From left to right, n = 31, 28, 83. **c**,**d**, Scheme representing exogenously expressed GFP-tagged Polθ. Representative images and quantification of GFP-Polθ (Polθ) foci following indicated treatment. In (c), from left to right, n = 90, 65, 32. In (d), from left to right, n = 120, 110, 100, 120. **e**, Quantification of Polθ foci formation following transfection with indicated siRNAs. From left to right, n = 982, 1082, 545, 45, 1152, 304, among (4, 4, 3, 2, 4, 2) replicates **f**, Representative images and quantification of IR-induced Polθ foci in HeLa cells expressing mAID-tagged (degron) BRCA2. BRCA2 degradation was achieved upon indole-3-acetic acid (IAA) treatment. At least 20 cells were analyzed in each condition in each replicate (two). **g**, Volcano plot of proteins co-immunoprecipitating with Polθ, as identified by label-free mass spectrometry. **h**, **i**, Immunoblot analysis following immunoprecipitation of (h) endogenous Neon Green (NG)-tagged Polθ and (i) exogenous GFP-tagged Polθ. **j**, Model for Polθ function and competition with HR in S phase. **k**, Panels showing representative images of Polθ foci formation in indicated phases of the cell cycle. Scale bars represent 5 μm. Data represents at least three biological replicates; except where indicated. For (b,f) unpaired t-test, for (c) Turkey’s multiple comparisons test, for (d,e) chi-square test, was performed. Chi-square statistics, in (d), from left to right: 24.8814, 12.9097; in (e), from left to right: 104.9835, 19.147, 401.2941, 78.6678.

To investigate the function of Polθ in human cells, we tagged the endogenous *POLQ* locus with a NeonGreen tag or exogenously expressed GFP-tagged Polθ in several human cell line models. In these cells, Polθ localized to sites of DSBs in a poly (ADP-ribose) polymerase (PARP)-dependent manner, as previously reported^24,25^ (Extended Data Fig. 1a–d). To decipher the regulation of Polθ localization to DSBs, we performed an unbiased immunofluorescence screening of Polθ foci formation. In brief, we first transfected RPE-1 cells expressing GFP–Polθ with a short interfering RNA (siRNA) library targeting factors involved in the DNA damage response (DDR). The cells were exposed to ionizing radiation and Polθ foci were scored by automatic fluorescence microscopy. As expected, siRNAs against *POLQ* or *FANCD2*^28^ abolished Polθ foci formation (Fig. 1a). Surprisingly, knockdown of genes encoding core factors in HR, such as *BARD1*, *BRCA1*, *PALB2* or *BRCA2* resulted in a marked reduction of Polθ foci formation (Fig. 1a and Supplementary Table 1). The results of the screen were validated by independent, siRNA or mAID (degron)-mediated knockdown of various HR proteins (Extended Data Fig. 1e,f). Additionally, immunoprecipitation coupled to label-free mass spectrometry analysis of tagged Polθ showed that Polθ co-purified with several members of the HR pathway, such as BARD1, BRCA1, PALB2 and BRCA2 (Extended Data Fig. 1g–i and Supplementary Table 2). Together, our data provide evidence that Polθ recruitment to DSBs is reliant on the HR pathway in interphase. These results are consistent with a role of Polθ in the repair of HR intermediates^29^, further suggesting crosstalk between Polθ and HR in S phase^24,30,31^ (Extended Data Fig. 1j).

## HR-independent Polθ recruitment in G2/M

**Extended Data Fig. 2:**
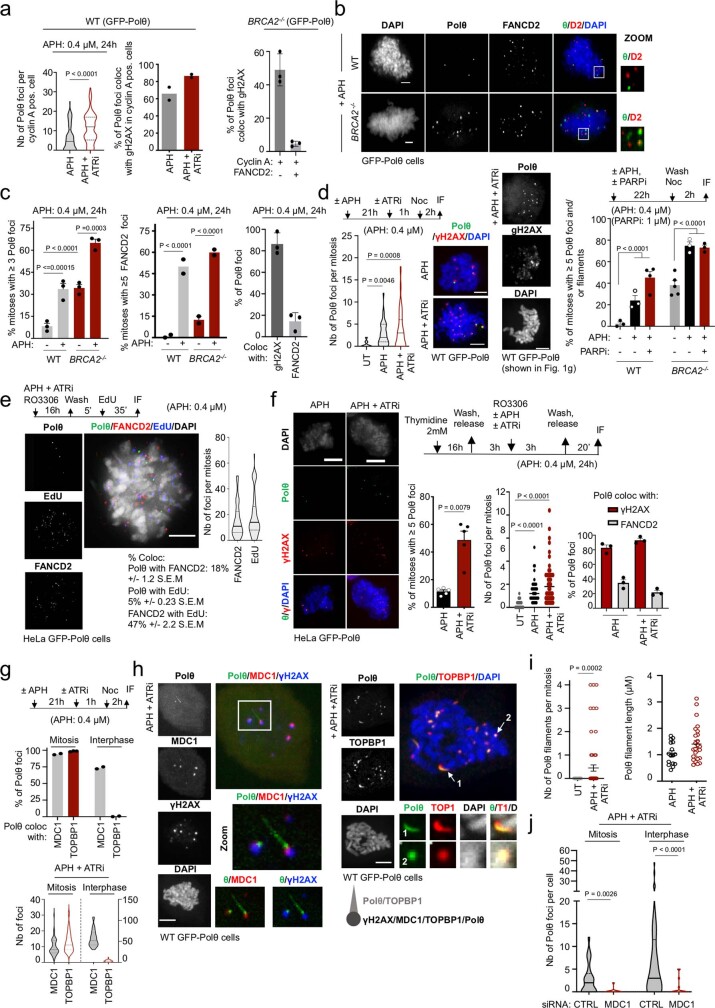
related to Fig. 1. Polθ forms replication stress (RS)-induced foci in mitosis. **a**,**b**,**c**,**d**, Representative images and quantification of exogenous GFP-Polθ foci formation in *WT* and *BRCA2*^−/−^ cells following indicated treatment. From left to right, in (a), n = 102, 112 (nb of cells); in (c), n = 90, 90, 91, 90; in (d-1 (left)), n = 20, 37, 65, among two replicates; in (d-2 (right)), at least 75 mitoses were analyzed for each condition in each replicate. **e**,**f**, Experimental design, representative images and quantification of Polθ foci (green) colocalization with DNA synthesis (EdU, blue) and FANCD2 (red) in mitotic cells. In (e), n = 62; in (f), from left to right, n = 41, 37, 44. **g**, Quantification of Polθ foci colocalization with MDC1 and TOPBP1. On top, n = 20 (cells), among two replicates; on bottom, from left to right, n = 50, 27, 36, 64 (cells), among two replicates. **h**, Representative images of Polθ foci and filament colocalization with γH2AX, MDC1 and TOPBP1 in mitosis. **i**, Quantification of Polθ filaments upon indicated treatment. From left to right, n = 26, 16, 26, 16. **j**, Quantification of Polθ foci formation upon MDC1 depletion in mitotic and interphase cells following indicated treatment. From left to right, n = 27, 11, 41, 16 among two replicates. Scale bars represent 5 μm, except (b), where 1 μm. Data represents at least three biological replicates. Data shows mean +/− S.E.M., except violin plots (g, j) showing median with quartiles. For (a,d, f-1 (left), i, j) two-tailed Mann-Whitney test; for (c) chi-square test, with chi-square statistics from left to right: 18.7626, 22.1553, 17.4308; for (f-2 (right)) Kruskal-Wallis test, corrected with Dunn’s multiple comparisons test was performed.

To decipher the conundrum of HR-dependent Polθ foci formation and synthetic lethality between Polθ and HR^24,25^, we compared Polθ foci formation in HR-deficient cells (*BRCA2*^*−/−*^) with that in wild-type isogenic counterparts^26^. We observed that the cell cycle distribution of Polθ foci in *BRCA2*^*−/−*^ cells was the inverse of that of wild-type cells. In wild-type cells, Polθ foci were visible mostly in S phase (PCNA-positive) cells, whereas in *BRCA2*^*−/−*^ cells, Polθ foci were prominent in G1 and G2 (PCNA-negative) cells but were absent in S (Fig. 1b, Extended Data Figs. 1k and 2a and Supplementary Videos 1–3). Together, our data show that whereas Polθ foci formation is dependent on HR in S phase, it is independent of HR in G2 and G1. This indicates a distinct, HR-independent function of Polθ from G2 to the subsequent G1 phase. Using live-cell microscopy, we found that in *BRCA2*^*−/−*^ cells, Polθ accumulated in G2, close to mitotic entry (after the disappearance of PCNA foci) (Fig. 1c). Additionally, Polθ foci in G2 could be induced by replication stress (aphidicolin treatment) in wild-type cells, to the level of that in *BRCA2*^*−/−*^ cells (Fig. 1d). Of note, the vast majority of Polθ foci in *BRCA2*^*−/−*^ cells were transmitted from G2 to mitosis (Fig. 1e). Together, our findings indicate that in *BRCA2*^*−/−*^ cells, Polθ marks intrinsic replication stress-induced lesions in G2 that remain unresolved and are transmitted to mitosis.

## Polθ is recruited to mitotic DSBs

Similar to G2 cells, mitotic cells showed an increase in Polθ foci formation upon replication stress, confirming the persistence of Polθ-marked lesions (Fig. 1f and Extended Data Fig. 2b–d). Almost all mitotic Polθ foci co-localized with phosphorylated histone H2AX (γH2AX, a DSB marker), indicating that Polθ marks replication stress-induced DSBs in mitosis (Fig. 1g and Extended Data Fig. 2c,d). Of note, Polθ co-localized poorly with mitotic DNA synthesis foci^32^ (labelled by EdU and FANCD2), indicating a mitotic DNA synthesis-independent role of Polθ (Extended Data Fig. 2e,f). Although HR and NHEJ repair are inactivated in mitosis, early events of DDR still occur, such as ATM-dependent recruitment of the scaffold proteins MDC1 and TOPBP1 to DSBs^11,33,34^. The vast majority of mitotic Polθ foci co-localized with MDC1 and TOPBP1 foci, whereas Polθ foci in interphase co-localized only with MDC1 but not with TOPBP1 (Extended Data Fig. 2g). Of note, Polθ also formed filament-like structures that associated with TOPBP1 (Fig. 1h and Extended Data Fig. 2h,i). This was particularly evident in cells under replication stress, suggesting that these mitotic filaments are key to the cellular response to replication stress (Extended Data Fig. 2h,i). Furthermore, knockdown of MDC1 prevented the formation of Polθ foci in both interphase and mitosis, whereas TOPBP1 knockdown suppressed Polθ foci (and filament) formation only in mitosis (Fig. 1i and Extended Data Fig. 2j). This shows that although the interaction between MDC1 and Polθ occurs similarly in both interphase and mitosis, the interaction between TOPBP1 and Polθ is specific to mitosis.

**Extended Data Fig. 3:**
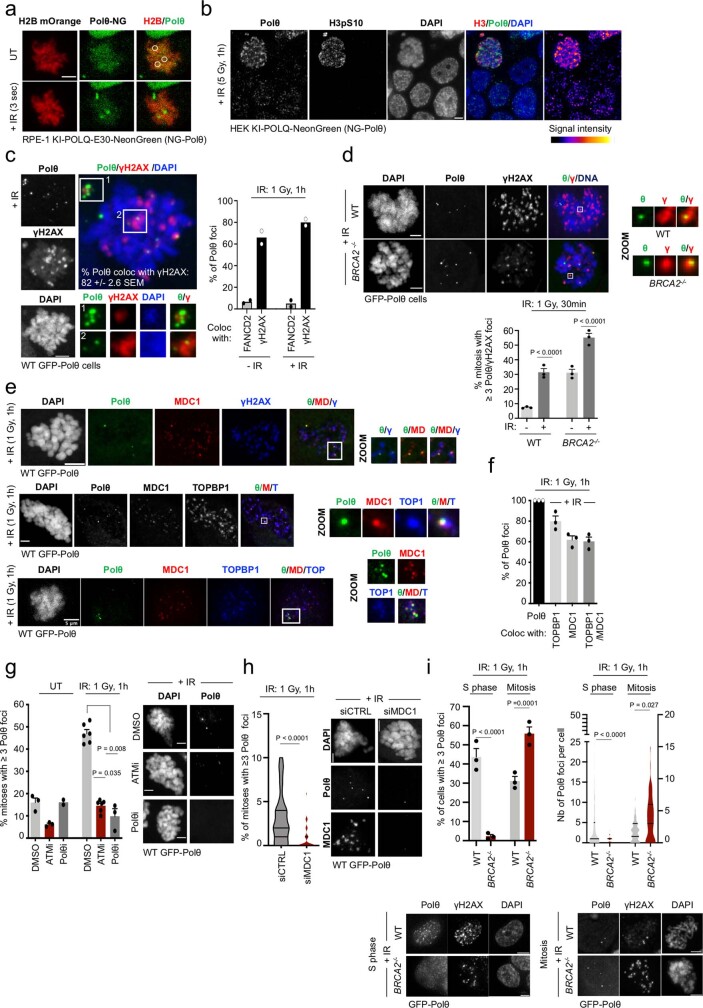
related to Fig. 1. Polθ forms radiation (IR)-induced foci in mitosis. **a**,**b**, Representative images of endogenous NG-Polθ foci formation upon (a) laser microirradiation or (b) X-ray irradiation (5 Gy, 1 h) in HEK cells. H3pS10 stains mitotic chromatin. **c**,**d**,**e**,**f**,**g**, Representative images and quantification of Polθ foci formation and colocalization with indicated proteins in mitosis following indicated doses of (X-ray) irradiation. In (d), from left to right, n = 88, 90, 76, 81; in (c,f), at least 35; in (g) at least 20 cells were analyzed in each condition in each replicate (two for (f)). **h**, Quantification of irradiation-induced Polθ foci following transfection with indicated siRNAs. From left to right, n = 57, 38, among two replicates. **i**, Representative images and quantification of irradiation-induced Polθ foci in WT and *BRCA2*^−/−^ cells in indicated cell cycle phases. From left to right, n = 974, 276, 75, 73. Scale bars represent 5 μm. Data represents three biological replicates, except where indicated. Data shows mean +/− S.E.M. For. For (d, i-2 (right)) chi-square test was performed, with chi-square statistics from left to right, in (d): 20.6912, 21.258; in (i-2): 160.6157, 9.7992; for (h) two-tailed Mann-Whitney test; for (g, i-1 (left)) Kruskal-Wallis test, corrected with Dunn’s multiple comparisons test was performed.

To further confirm the recruitment of Polθ to mitotic DSBs, we irradiated mitotic cells (collected by gentle shaking off of nocodazole-arrested cells) and measured Polθ foci formation. Polθ foci formation could be induced by ionizing radiation (Extended Data Fig. 3a–d), consistent with recent findings reporting Polθ localization to mitotic DSBs^19,20^. Furthermore, ionizing radiation-induced mitotic Polθ foci (similarly to those induced by replication stress) co-localized with the TOPBP1–MDC1 complex and their formation was dependent on ATM and MDC1 (Extended Data Fig. 3e–i). Together, these results suggest that whereas the early steps of the DDR mediated by ATM and MDC1 are conserved between interphase and mitosis, repair pathways diverge downstream of MDC1. Classical DSB repair (HR and NHEJ) dominates in interphase, whereas a TOPBP1- and Polθ-mediated mitotic repair pathway responds to mitotic DSBs.

## PLK1 phosphorylates Polθ in mitosis

**Extended Data Fig. 4:**
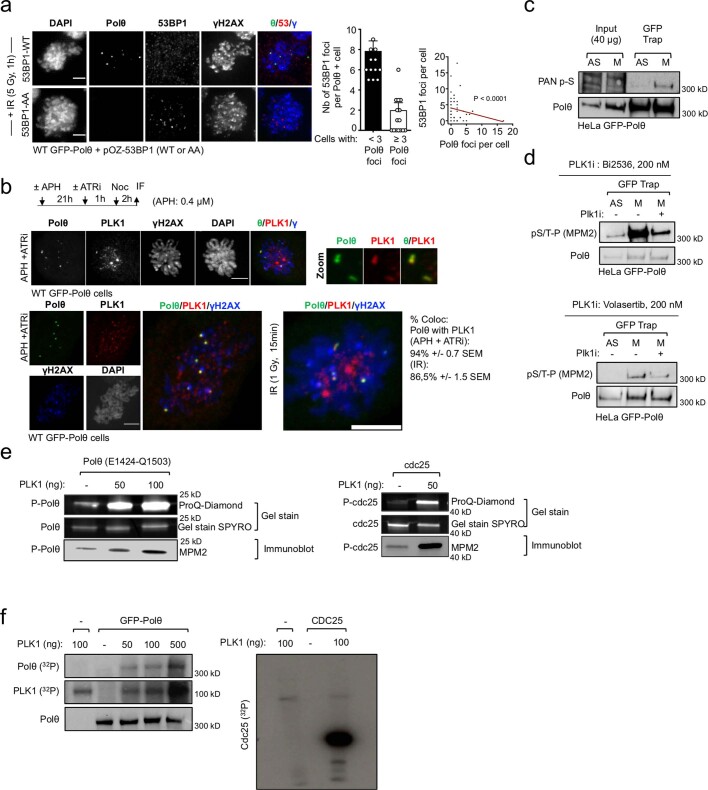
related to Fig. 2. Polθ is phosphorylated by PLK1 in mitosis. **a**, Representative images and quantification of GFP-Polθ foci formation in cells expressing wild-type (WT) or mutant (AA) 53BP1 upon irradiation in mitosis. 30 cells were analyzed among 3 replicates. Data shows mean +/− S.E.M. Fisher’s exact test was performed to determine significance. **b**, Representative images and quantification of Polθ foci colocalization with PLK1 in mitosis following indicated treatment. Experiments were repeated twice with similar results. **c**,**d**, Immunoblot analysis following immunoprecipitation of GFP-tagged Polθ in asynchronous or mitotic cells upon indicated treatment. Representative experiments are shown; experiments were repeated three times with similar results. **e**, Representative *in vitro* phosphorylation assay with the Polθ fragment (E1424 to Q1503) incubated with recombinant PLK1 enzyme. Phosphorylation signals were revealed using Gel Stain and immunoblot analyses (MPM2 antibody). Cdc25 phosphorylation by PLK1 is used as a control. Representative experiments are shown; experiments were repeated twice with similar results. **f**, Representative *in vitro* kinase assay with Polθ incubated with recombinant PLK1 enzyme and ^32^P-labeled ATP. Representative experiment is shown; experiment was repeated three times with similar results.

We next sought to explain the regulation of mitotic Polθ activity. The mitotic kinases CDK1 and PLK1^35^ restrict classical DSB repair through the phosphorylation of several NHEJ and HR factors such as 53BP1 and BRCA2^3,4,36^. A 53BP1 mutant (T1609A/S1618A) that cannot be phosphorylated by PLK1 escapes negative regulation and forms ionizing radiation-induced foci in mitosis^4^. Notably, this unrestrained 53BP1 foci formation abolished Polθ foci formation in mitosis (Extended Data Fig. 4a). We also observed a colocalization between Polθ and PLK1 foci (Extended Data Fig. 4b). This suggests a competition between the two pathways (NHEJ and Polθ-mediated end joining), with PLK1 as a mediator of pathway choice. This notion prompted us to test for the potential phosphorylation of Polθ by PLK1 in mitosis.

**Fig. 2:**
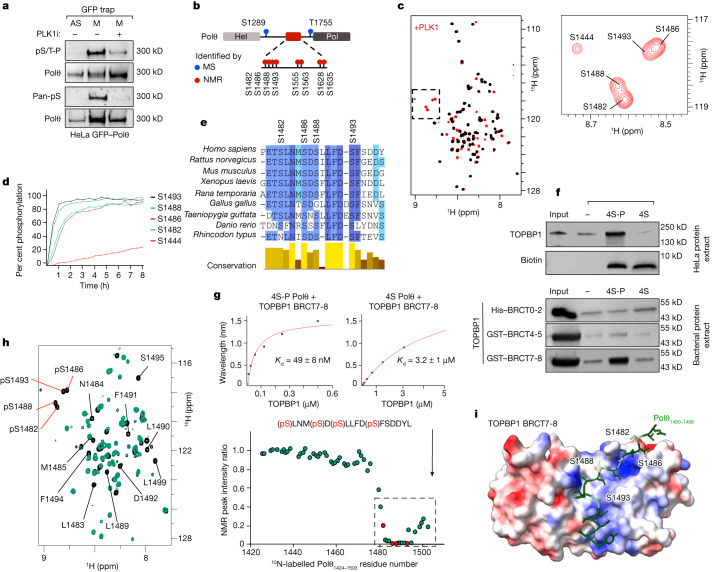
Polθ is phosphorylated by PLK1 and binds to TOPBP1 in mitosis. **a**, Immunoblot analysis with anti-Polθ, anti-phospho-Ser/Thr-Pro (pS/T-P) and pan-phosphoserine antibody following immunoprecipitation of Polθ–GFP from asynchronous (AS) and mitotic (M) cells. A representative experiment is shown; the experiment was repeated four times with similar results. **b**, Scheme depicting PLK1 phosphorylation sites on Polθ. MS, mass spectrometry. **c**, Left, superposition of the 2D NMR ^1^H-^15^N selective optimized flip angle short transient heteronuclear single quantum coherence (SOFAST HMQC) spectra recorded on an ^15^N,^13^C-labelled Polθ E1424–Q1503 fragment before (black) and after (red) incubation with PLK1. Right, magnified view of the spectral region containing NMR signals of phosphorylated residues. **d**, Phosphorylation kinetics, as monitored by real-time NMR. Intensities of NMR peaks corresponding to phosphorylated residues shown in **c** were measured on a series of SOFAST HMQC spectra recorded on the Polθ E1424–Q1503 fragment incubated with PLK1 and plotted as a function of time. **e**, Alignment and conservation score of nine homologous sequences of Polθ from vertebrates. Colours reflect the conservation score. **f**, Immunoblot analysis following immunoprecipitation of phosphorylated (4S-P) and non-phosphorylated Polθ peptides incubated with HeLa cell protein extract (top), and indicated TOPBP1 BRCT domains purified from *E. coli* (bottom). A representative experiment is shown; the experiment was repeated twice with similar results. **g**, Binding kinetics of phosphorylated (4S-P) and non-phosphorylated (4S) Polθ peptide titrated with purified TOPBP1 BRCT7-8, as observed by BLI. *K*_d_, dissociation constant. **h**, Left, superposition of the 2D NMR ^1^H-^15^N SOFAST HMQC spectra recorded on a ^15^N-labelled phosphorylated Polθ E1424–Q1503 fragment before (black) and after (green) incubation with TOPBP1 BRCT7-8. Phosphorylation sites are indicated by red lines. Right, plot of the intensity ratio of peaks corresponding to each residue (in the bound versus free peptide) as a function of Polθ residue number. The dashed line outlines NMR peaks that disappear owing to the interaction of Polθ with TOPBP1 BRCT7-8. Red dots indicate phosphorylated residues. **i**, Model of the Polθ–TOPBP1 interaction, calculated using AlphaFold-Multimer. The Polθ peptide is displayed as green sticks, with phosphorylated serines in pale green. The TOPBP1 BRCT7-8 domain is displayed as a surface coloured as a function of its electrostatic potential (red, negatively charged; blue, positively charged).

To investigate this, we immunoprecipitated Polθ and assessed its phosphorylation by immunoblot analysis using pan-phospho antibodies. We observed Polθ phosphorylation in mitosis, but not in interphase cells, suggesting that Polθ is phosphorylated mainly in mitosis (Fig. 2a and Extended Data Fig. 4c,d). This phosphorylation was markedly reduced when cells were treated with two different PLK1 inhibitors (PLK1i), indicating that PLK1 is responsible for Polθ phosphorylation in mitosis (Fig. 2a and Extended Data Fig. 4d). Furthermore, in vitro incubation of Polθ immunoprecipitates with purified recombinant PLK1 enzyme in the presence of radioactive ATP confirmed a direct phosphorylation of Polθ by PLK1 (Extended Data Fig. 4f).

**Extended Data Fig. 5:**
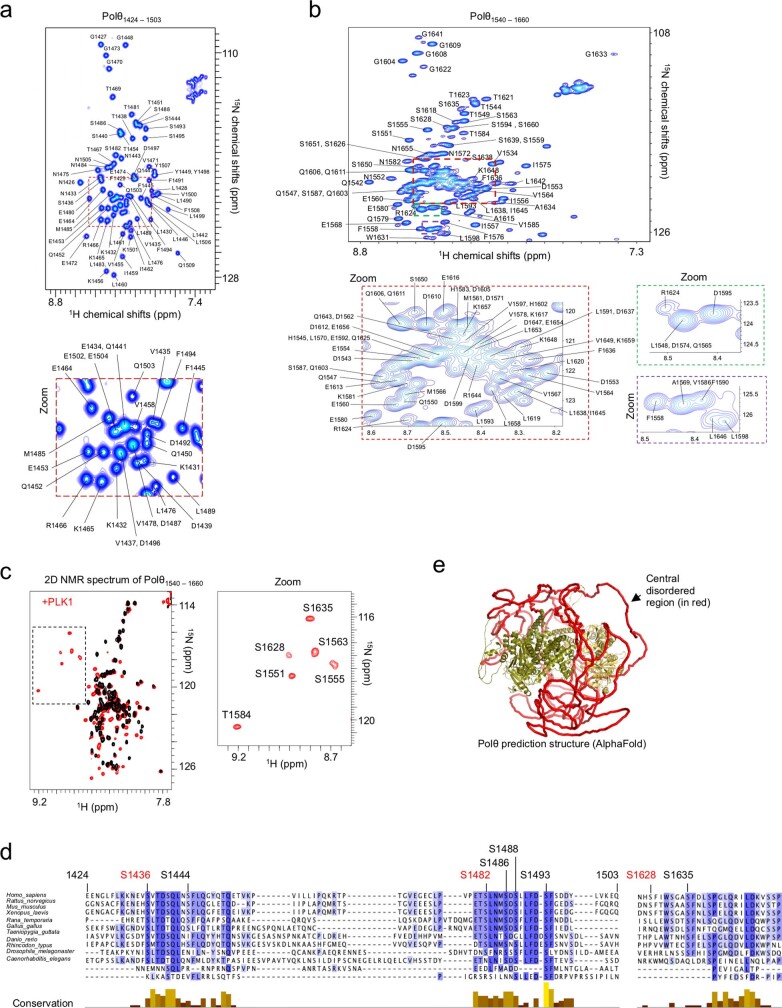
related to Fig. 2. Biochemical characterization of Polθ phosphorylation by PLK1. **a**,**b**, Assignment of the 2D NMR ^1^H-^15^N SO-FAST HMQC peaks of the indicated Polθ fragments (E1424-Q1503 for a) (E1540-S1660 for b). **c**, Superposition of the 2D NMR ^1^H-^15^N SO-FAST HMQC spectra recorded on an ^15^N, ^13^C labeled Polθ fragment from E1540 to S1660, before (black) and after (red) incubation with PLK1. A zoom shows the spectral region containing the NMR signals of phosphorylated residues. **d**, Alignment of 11 homologous sequences of the Polθ fragment E1424-Q1503 and N1626-P1652. All labeled residues were detected as phosphorylated by PLK1. Residues marked in red correspond to canonical PLK1 phosphorylation sites. The conservation score was calculated using Jalview. **e**, AlphaFold model of the human Polθ protein. The two folded N-terminal and C-terminal domains are colored in green and yellow, respectively, while the disordered central region, from G895 to S1824, is colored in red.

To identify the Polθ residues phosphorylated by PLK1, we performed quantitative mass spectrometry-based phosphorylation analysis of Polθ in mitosis, in the presence or absence of PLK1i. This analysis identified several potential PLK1 phosphorylation sites on Polθ, including several predicted PLK1 phosphorylation sites^37,38^ (Fig. 2b and Supplementary Tables 4 and 5). To confirm these sites, we applied NMR spectroscopy to two recombinant fragments purified from *Escherichia coli* (Polθ E1424–Q1503 and Polθ E1540–S1660), which contain most of the predicted sites. We identified eight PLK1 phosphorylation sites with high confidence (Fig. 2c, Extended Data Fig. 5a–c and Supplementary Tables 4 and 5). The fast kinetics of Polθ phosphorylation observed by NMR suggests that Polθ is a preferential substrate for PLK1 (Fig. 2d). Two of the sites identified by mass spectrometry were outside of the two fragments analysed by NMR; we thus identified a total of ten PLK1 phosphorylation sites on Polθ. All ten phosphorylation sites are evolutionarily conserved, even though they are located within the large, non-conserved, central region of Polθ. Four of the phosphorylation sites (S1482, S1486, S1488 and S1493) are clustered in a region that is highly conserved among vertebrates (E1480–F1494), indicating that these residues have a crucial function (Fig. 2e, Extended Data Fig. 5d and Supplementary Table 5).

## Polθ binds to TOPBP1 in mitosis

**Extended Data Fig. 6:**
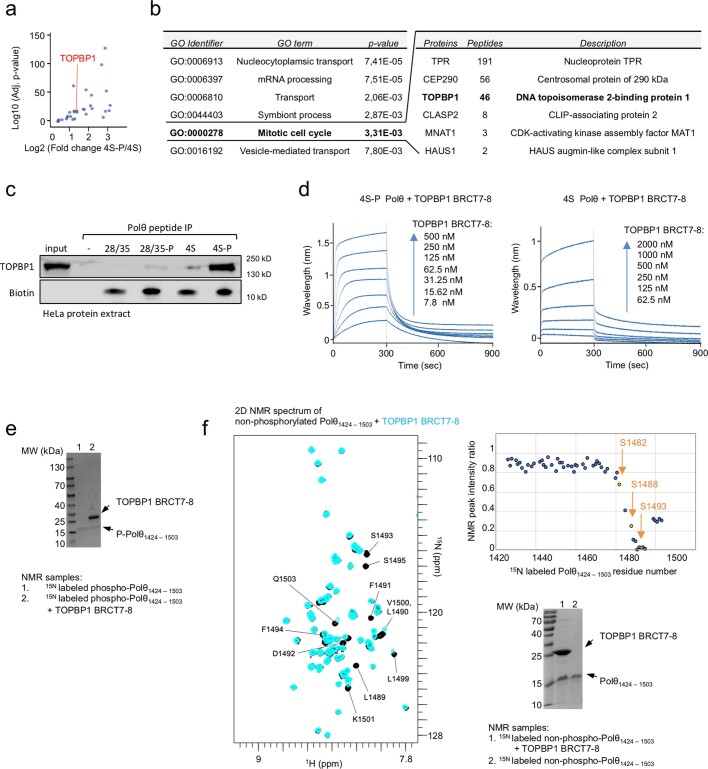
related to Fig. 2. Characterization of Polθ binding to TOPBP1. **a**, Volcano plot of proteins co-immunoprecipitating with 4S-P Polθ peptide, as identified by label-free mass spectrometry. **b**, Enriched GO terms of biological processes, identified among the most enriched binding partners of the 4S-P Polθ peptide as compared to the 4S Polθ. **c**, Immunoblot analysis following immunoprecipitation of indicated phosphorylated and non-phosphorylated Polθ peptides incubated with HeLa cell protein extracts. Representative experiment is shown; experiment was repeated twice with similar results. **d**, Association and dissociation curves of indicated Polθ peptide (S4-P and 4S) in the presence of increasing TOPBP1 BRCT7-8 concentrations, as observed by Bio-layer interferometry. **e**, SDS-PAGE of the indicated NMR samples for the experiments shown in 2h. Representative experiment is shown; experiment was repeated twice with similar results. **f**, Superposition of the 2D NMR ^1^H-^15^N SO-FAST HMQC spectra recorded on an ^15^N-labeled non-phosphorylated Polθ fragment from Glu1424 to Gln1503, before (black) and after (blue) incubation with TOPBP1 BRCT7-8. The intensity ratio of the peaks corresponding to each residue (in the bound versus free peptides) is plotted as a function of the Polθ residue number. A box marks the NMR peaks disappearing due to the Polθ interaction with TOPBP1 BRCT7-8. The SDS-PAGE of the NMR samples is shown. Representative experiment is shown; experiment was repeated twice with similar results.

TOPBP1 contains nine BRCA1 C-terminal (BRCT) domain repeats, several of which selectively interact with phosphorylated peptides^39^. We have shown that TOPBP1 controls Polθ recruitment to mitotic DSBs. Furthermore, all identified phosphorylation sites on Polθ are within a region that is disordered (as predicted by AlphaFold^40^), and thus accessible for mediating protein–protein interactions (Extended Data Fig. 5e). On the basis of these observations, we hypothesized that TOPBP1 recruits Polθ in mitosis by direct binding through its BRCT domains. To test this hypothesis, we compared binding partners of phosphorylated (4S-P) and non-phosphorylated (4S) Polθ peptides (E1472–Y1498) containing the 4 clustered serines (S1482, S1486, S1488 and S1493). Quantitative mass spectrometry identified TOPBP1 as one of the most enriched proteins that bind specifically to the phosphorylated Polθ peptide (Extended Data Fig. 6a,b). Phosphorylation-dependent binding of TOPBP1 to Polθ was confirmed by Polθ peptide immunoprecipitation followed by immunoblot analysis (Fig. 2f). A Polθ peptide containing two phosphorylated serines outside of the four clustered ones (S1628 and S1635) did not interact with TOPBP1, suggesting a highly specific interaction between the four clustered serines and TOPBP1 (Extended Data Fig. 6c). To further specify the interaction between Polθ and TOPBP1, we purified the three phosphopeptide-binding BRCT domains of TOPBP1 (namely, BRCT0-2, BRCT4-5 and BRCT7-8) from *E. coli* and tested their interaction with Polθ peptides in vitro. We found that phosphorylated Polθ (4S-P) binds directly to the BRCT7-8 domain of TOPBP1(Fig. 2f).

Furthermore, bio-layer interferometry (BLI) revealed that phosphorylated Polθ (4S-P) has a 65-fold higher affinity for TOPBP1 BRCT7-8 compared with non-phosphorylated Polθ (4S) (Fig. 2g and Extended Data Fig. 6d). We recorded 2D NMR spectra of a phosphorylated recombinant Polθ fragment (E1424–Q1503) before and after incubation with purified TOPBP1 BRCT7-8 domain. NMR signals corresponding to the Polθ region (p-S1482–L1499) were lost upon addition of BRCT7-8 (Fig. 2h and Extended Data Fig. 6e). This indicates that the Polθ region containing the four clustered serines (S1482–L1499) is located at the interface with TOPBP1 in the complex. Of note, a similar NMR analysis using the non-phosphorylated recombinant Polθ (E1424–Q1503) revealed that only a few NMR peaks were lost upon addition of TOPBP1 BRCT7-8 (Extended Data Fig. 6f). This indicates that the interface between Polθ and TOPBP1 is significantly reduced in the absence of phosphorylation by PLK1. This is consistent with the weaker affinity of non-phosphorylated Polθ for TOPBP1 compared with phosphorylated Polθ, as measured by BLI. Finally, we generated a series of models for the interaction using AlphaFold^40^. We observed that the four clustered serines (S1482, S1486, S1488 and S1493) interact with a positively charged surface of TOPBP1 BRCT7-8 (Fig. 2i).

Thus, mitotic phosphorylation of Polθ by PLK1 regulates its interaction with TOPBP1. This interaction is mediated by direct binding of a cluster of four phosphorylated serines in the disordered central domain of Polθ to the BRCT7-8 domain of TOPBP1.

## Polθ repairs mitotic DSBs

**Fig. 3:**
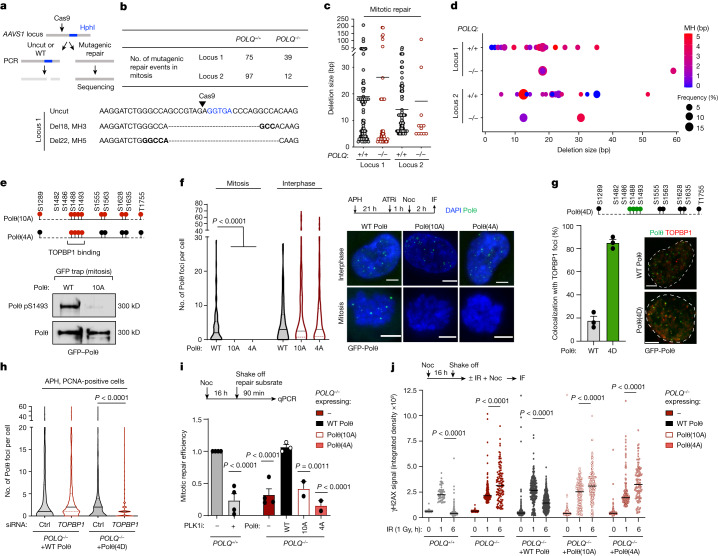
Polθ phosphorylation and interaction with TOPBP1 enable DSB repair in mitosis. **a**, Experimental workflow. **b**, Top, the number of mitotic DNA repair events identified following CRISPR–Cas9-induced cleavage in indicated cell lines. Bottom, representative repaired DNA sequences. Deletions (Del) and microhomology (MH) sizes are indicated. At repair junctions, microhomologies are indicated in bold and the Hph1 recognition site is in blue. **c**, Deletion size of mitotic DNA repair events identified in wild-type and *POLQ*^−/−^ cells. Each dot represents a mitotic DNA repair event. **d**, The frequency, deletion size and use of microhomology in mitotic DNA repair events identified in wild-type and *POLQ*^−/−^ cells. Events with deletions size greater than 60 bp are represented. **e**, Top, schematic representation of phosphorylation sites mutated in Polθ(10A) and Polθ(4A) constructs. Bottom, immunoblot analysis following immunoprecipitation of indicated construct. **f**, Representative images and quantification of Polθ foci and filaments in cells expressing wild-type Polθ, Polθ(10A) and Polθ(4A). Left to right: *n* = 98, 60, 37, 74, 219 and 50. **g**, Top, schematic representation of phosphorylation sites mutated in Polθ(4D). Bottom, representative images (right) and quantification (left) of Polθ foci colocalizing with TOPBP1 in cells expressing wild-type Polθ and Polθ(4D). Wild-type Polθ: *n* = 180; Polθ(4D): *n* = 150. **h**, Quantification of Polθ foci formation in cells expressing wild-type Polθ and Polθ(4D) upon indicated treatment in S phase cells. WT Polθ control: *n* = 620; WT Polθ *TOPBP1*: *n* = 582; Polθ(4D) control: *n* = 441; Polθ(4D) *TOPBP1*: *n* = 880. **i**, Mitotic repair efficiency in indicated cell lines upon indicated treatment. Left to right: *n* = 4, 4, 4, 3, 2 and 2. **j**, Quantification of γH2AX signal at different time points after ionizing radiation (1 Gy) in mitosis. Left to right: *n* = 119, 59, 152, 81, 203, 188, 388, 227, 532, 181, 155, 239, 216, 159 and 102, with two replicates. Mixed-effects analysis, corrected with Holm–Šídák’s multiple comparisons test. Scale bars, 5 μm. Data represent three biological replicates, except where indicated. Data are mean ± s.e.m., except in violin plots (**e**), which show median with quartiles. **f**,**h**,**j**, Kruskal–Wallis test, corrected with Dunn’s multiple comparisons test.

**Extended Data Fig. 7:**
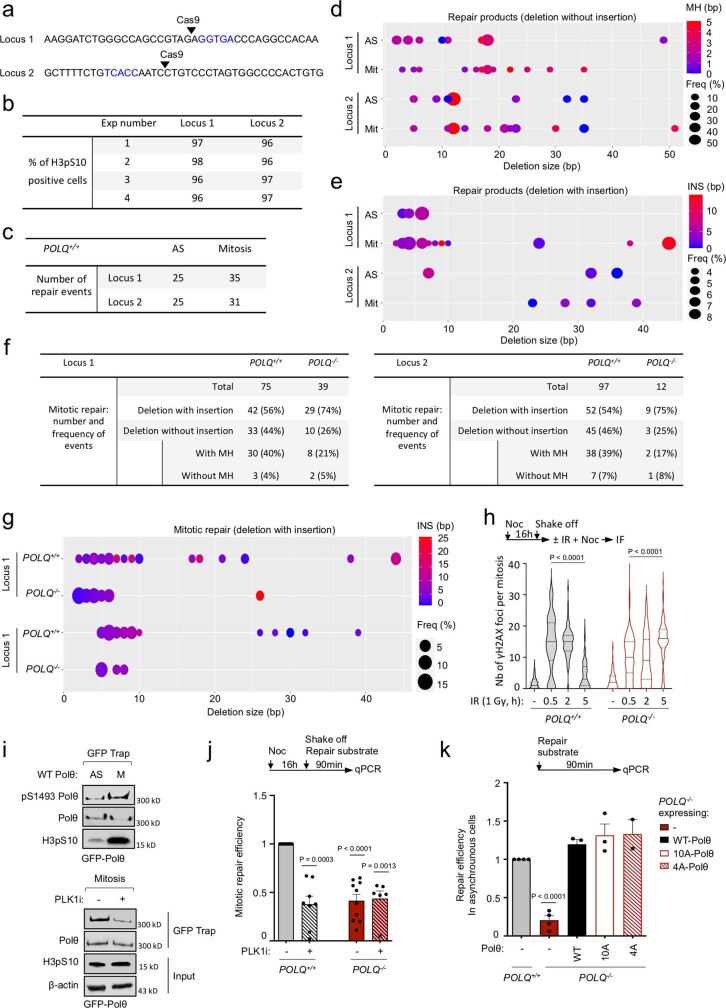
related to Fig. 3. Polθ repair signature in asynchronous and mitotic cells. **a**, DNA sequences of CRISPR-Cas9 induced-cleavage sites at locus 1 and locus 2. Blue letters indicate Hph1 recognition site. **b**, Quantification of H3pS10 positive cells (mitotic), assessed by immunofluorescence, in indicated experiments. **c**, Total number of DNA repair events obtained at both loci from asynchronous (AS) and mitotic cells. **d**,**e**, Graph showing the frequency, deletion size and MH use of DNA repair events identified in asynchronous (AS) and mitotic (Mit) cells. **f**, Table recording number and frequency of repair events (with or without insertion). **g**, Graph showing the frequency, deletion and insertion size of mitotic DNA repair events identified in WT and *POLQ*^−/−^ cells. **h**, Quantification of γH2AX signal at different time points after radiation (1 Gy) in mitosis. From left to right, n = 119, 60, 91, 104, 81, 203, 91, 116. **i**, Immunoblot analyses following immunoprecipitation of indicated constructs. **j**,**k**, DNA repair efficiency upon indicated treatment and in mitotic (j) or asynchronous (k) indicated cell lines. From left to right, for (j), n = 10, 8, 10, 6; for (k), n = 4, 4, 3, 3, 2. Data represent three biological replicates, except where indicated. Data shows mean +/− S.E.M., expect violin plots (i) showing median with quartiles. For (h) Kruskal-Wallis test, corrected with Dunn’s multiple comparisons test; for (j,k) mixed-effects analysis was performed, corrected with Holm-Šídák’s multiple comparisons test.

In mitosis, although it is widely accepted that HR and NHEJ repair activities are inhibited, the activity of alternative pathways remains largely unaddressed. Although there has not been direct evidence of DSB repair in mitosis, recent data suggests that alternative end joining^15,16^ and Polθ could be active in mitosis^17–20^. To detect DSB repair in mitosis, we took advantage of recent advances in genome editing that enable the induction of DSBs after Cas9 protein transfection. We transfected nocodazole-arrested cells with a Cas9–guide RNA (gRNA) complex to induce DSB formation at two different sites in the AAVS1 safe locus, whose mutagenic repair would destroy a nearby HphI restriction site. After DSB induction, a small genomic region around the cut site was PCR amplified and digested by HphI. Finally, undigested PCR products (representing mutagenic repair) were analysed by Sanger sequencing (Fig. 3a and Extended Data Fig. 7a).

We found evidence of mutagenic mitotic DSB repair at both of the loci that we tested (Fig. 3b). To rule out contamination from interphase cells, the purity of mitotic samples was confirmed by histone H3 phospho-S10 immunofluorescence (H3pS10) (a specific marker of mitosis) (96–100% purity) (Extended Data Fig. 7b). We next compared mitotic DSB repair with DSB repair in asynchronous control cells. Repair products obtained from cells in mitosis differed from those from asynchronous cells in that there were more products with deletions larger than 10 bp associated with the use of microhomologies, further ruling out the possibility of a contamination effect (Extended Data Fig. 7c–e).

Furthermore, we observed a marked difference in mitotic DSB repair between wild-type and *POLQ*^*−/−*^ cells (Fig. 3b–d, Extended Data Fig. 7f,g and Supplementary Table 3). The number of repair events was greatly diminished at both loci in *POLQ*^*−/−*^ cells, indicating that Polθ is responsible for mutagenic repair of mitotic DSBs (Fig. 3b). Previous studies have demonstrated that Polθ-mediated repair makes use of microhomologies and generates deletions of up to 60 bp^41–44^. Accordingly, we found that, whereas both wild-type and *POLQ*^*−/−*^ cells exhibited repair products with deletions smaller than 10 bp or larger than 60 bp in size, repair products with deletions in the 10- to 60-bp range were absent in *POLQ*^*−/−*^ cells (Fig 3c,d). In addition, we showed that the use of microhomologies was common in wild-type cells, but greatly diminished in *POLQ*^*−/−*^ cells (Extended Data Fig. 7f). Finally, to assess the contribution of Polθ to mitotic DSB repair in a more quantitative manner, we determined the kinetics of γH2AX foci resolution after ionizing radiation in mitosis. We found that, whereas the vast majority of γH2AX foci in wild-type cells were resolved 5 h after exposure to ionizing radiation, they persisted in *POLQ*^*−/−*^ cells (Extended Data Fig. 7h). Together, our results show that Polθ has a major role in mitotic end-joining repair. Rather than being an alternative repair factor in interphase, we posit that Polθ is the main DSB repair factor in mitosis.

## PLK1 controls mitotic DSB repair by Polθ

**Extended Data Fig. 8:**
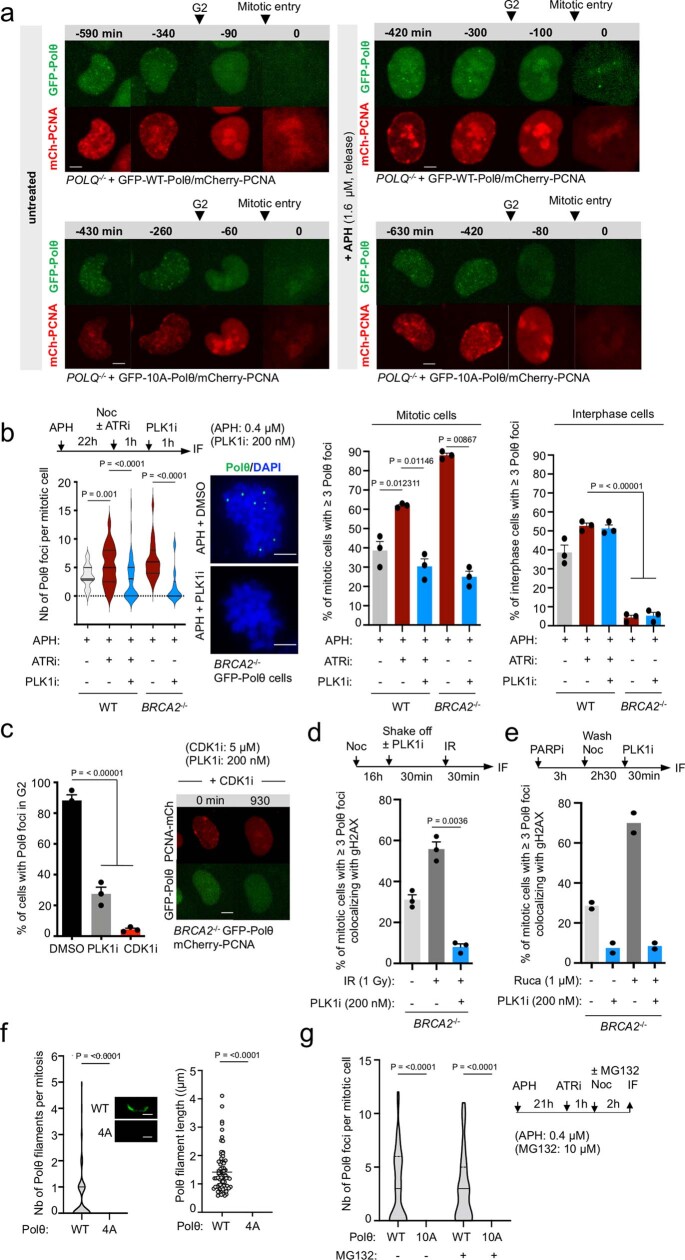
related to Fig. 3. Polθ foci formation in mitosis are dependent on its phosphorylation by PLK1. **a**, Representative images of live microscopy analysis of *POLQ*^*−/−*^ cells expressing WT- or 10A- Polθ and mCherry-PCNA. **b**,**c**,**d**,**e**, Representative images and quantification of Polθ foci formation following indicated treatment in indicated phases of the cell cycle in WT and *BRCA2*^*−/−*^ cells. In (b), from left to right, n = 46, 57, 57, 146, 40, 77, 95, 52, 150, 48, 203, 199, 156, 325, 152. In (c, d), n > 30 for each condition in each replicate. In (e), from left to right n = 52, 54, 63, 76; among two replicates. **f**, Quantification of number and length of Polθ filaments in cells expressing WT- or 4A-Polθ. From left to right, n = 87, 114, 77, 0. **g**, Quantification of Polθ foci formation following indicated treatment in cells expressing WT- or 10A-Polθ. Data represents two biological replicates. From left to right, n = 45, 24, 39, 37. Scale bars represent 5 μm. Data represents three biological replicates, except where indicated. Data shows mean +/− S.E.M., except violin plots (b,f,g) showing median with quartiles. For (b1 (left), g) Kruskal-Wallis test, corrected with Dunn’s multiple comparisons test; for b2-3, chi-square test, with chi-square statistics, from left to right: 6.2656, 10.5753, 6.8895, 95.0355, 45.1237; for (c) Mixed-effects analysis, corrected with Holm-Šídák’s multiple comparisons test; for (d) unpaired t-test; for (f) two-tailed Mann-Whitney test was performed.

To assess the functional consequences of Polθ phosphorylation by PLK1, we complemented *POLQ*^*−/−*^ cells with either wild-type Polθ or mutant versions of Polθ that cannot be phosphorylated. In Polθ(10A), all the identified phosphorylation sites are mutated to alanine, whereas in Polθ(4A) the cluster of four serines that are crucial for TOPBP1 binding are replaced with alanine (Fig. 3e and Supplementary Table 5). A phospho-specific antibody generated against the PLK1-phosphorylated site pS1493 confirmed mitotic phosphorylation of wild-type Polθ but not Polθ(10A) (Fig. 3e and Extended Data Fig. 7i). Although both Polθ(10A) and Polθ(4A) were recruited to replication stress-induced DSBs in interphase, neither mutant was recruited to DSBs in mitosis (Fig. 3f, Extended Data Fig. 8a and Supplementary Video 4), similar to our observations in wild-type Polθ expressing cells under PLK1 inhibition (Extended Data Fig. 8b–g). Of note, the phosphomimetic mutant Polθ(4D), in which all four clustered serines mediating the interaction with TOPBP1 are mutated to aspartic acid, can bypass PLK1 mitotic regulation and force TOPBP1–Polθ interaction in S phase (Fig. 3g,h). Together, our findings indicate that the recruitment of Polθ to mitotic DSBs is regulated by the phosphorylation-mediated interaction between Polθ and TOPBP1.

To evaluate the role of Polθ phosphorylation in mitotic DSB repair, we performed an in vitro end-joining repair assay^29^. In brief, cells arrested in mitosis were transfected with a DNA repair substrate containing microhomologies, and end-joining efficiency was measured by quantitative PCR (qPCR). Using this assay, we first confirmed that end-joining repair was active in mitosis and dependent on Polθ, as we have previously observed (Fig. 3i). In addition, PLK1 inhibition greatly impaired the efficiency of mitotic end joining, pointing towards a positive regulatory role for PLK1 in Polθ-mediated mitotic end-joining repair (Fig. 3i and Extended Data Fig. 7j). Confirming this, we found that expression of Polθ(10A) or Polθ(4A) did not rescue mitotic repair defects in *POLQ*^*−/−*^ cells, whereas defects in interphase cells were rescued (Fig. 3i and Extended Data Fig. 7k). Furthermore, expression of wild-type Polθ but not Polθ(10A) or Polθ(4A) rescued the accumulation of mitotic DSBs in *POLQ*^−/−^ cells, as assessed by the kinetics of γH2AX foci resolution after ionizing radiation (Fig. 3j). Together, our data show that PLK1 positively regulates mitotic end-joining repair, contrary to its inhibitory role on HR and NHEJ. PLK1-mediated phosphorylation of Polθ enables its recruitment to mitotic DSBs through its interaction with TOPBP1, facilitating subsequent end-joining repair.

## Polθ forms nuclear bodies in G1

**Fig. 4:**
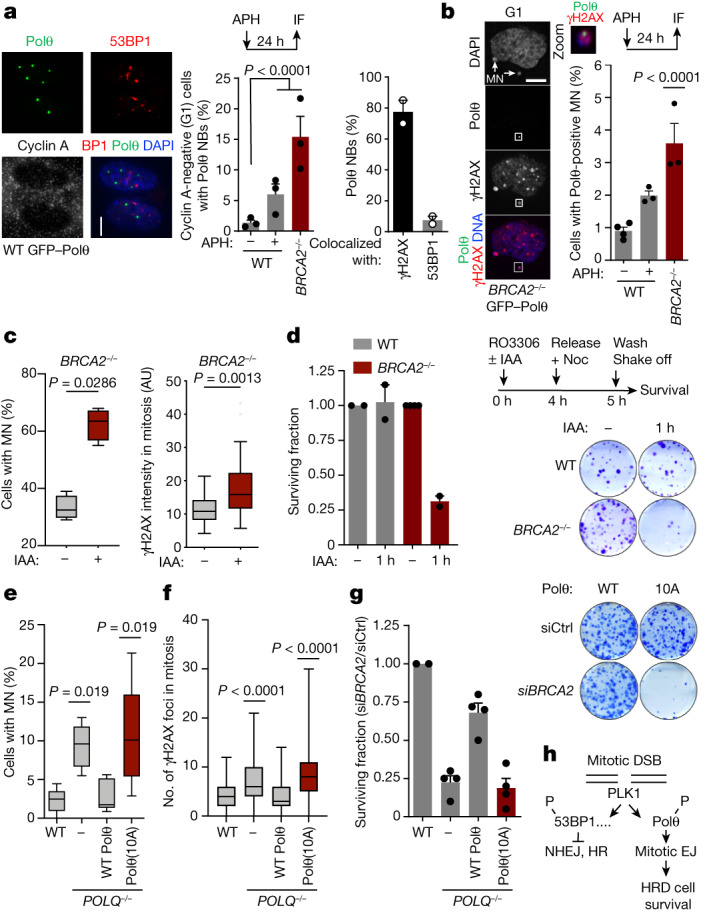
Polθ function in mitosis maintains genome stability and survival of HR-deficient cells. **a**, Representative images (left) and quantification (centre) of Polθ nuclear bodies (NBs) in cyclin A-negative (G1) cells. WT −aphidicolin: *n* = 362; WT +aphidicolin: *n* = 137; *BRCA2*^*−/−*^: *n* = 127. Right, colocalization of Polθ nuclear bodies with indicated proteins. At least 30 nuclear bodies were scored for each experiment. Scale bar, 5 μm. **b**, Representative images (left) and quantification (right) of Polθ-positive micronuclei (MN). White square indicates the enlarged section. WT −aphidicolin: *n* = 815; WT +aphidicolin: *n* = 448; *BRCA2*^*−/−*^: *n* = 552. Scale bar, 10 μm. **c**, Quantification of micronuclei (left, *n* = 4 replicates) and γH2AX intensity (right, *n* = 2 replicates) upon Polθ depletion in mitosis. Polθ depletion is achieved by indole-3-acetic acid (IAA) treatment (degron expression). Left to right: *n* = 89, 83, 31 and 43. AU, arbitrary units. **d**, Experimental workflow (top right), representative images (bottom right) and quantification (left) of 14-day clonogenic survival assays upon depletion of Polθ (IAA treatment) in mitosis. For wild type, the number of replicates is 2. **e**–**g**, Quantification of micronuclei (**e**), γH2AX foci (**f**) and clonogenic survival (**g**) in *POLQ*^−/−^ cells complemented with wild-type Polθ or Polθ(10A). **e**, Left to right: *n* = 6, 8, 6 and 7 replicates, with at least 100 cells analysed per condition for each experiment. **f**, Left to right: *n* = 120, 109, 104 and 123. **g**, Left to right: 2, 4, 4 and 4 replicates. siCtrl, control siRNA targeting LacZ; si*BRCA2*, siRNA targeting *BRCA2*. **h**, Model for the function of Polθ in mitosis. EJ, end joining. Data represent three biological replicates, except where indicated. Data are mean ± s.e.m. except in box plots (**c**,**e**,**f**), which show median with minimum and maximum values. **a**,**b**, Chi-square test. **a**, Aphidicolin, χ^2^ = 6.8345; *BRCA2*^*−/−*^, χ^2^ = 33.9998. **b**, χ^2^ = 14.2308. **c**, Two-tailed Mann–Whitney test. **e**,**f**, Kruskal–Wallis test, corrected with Dunn’s multiple comparisons test.

**Extended Data Fig. 9:**
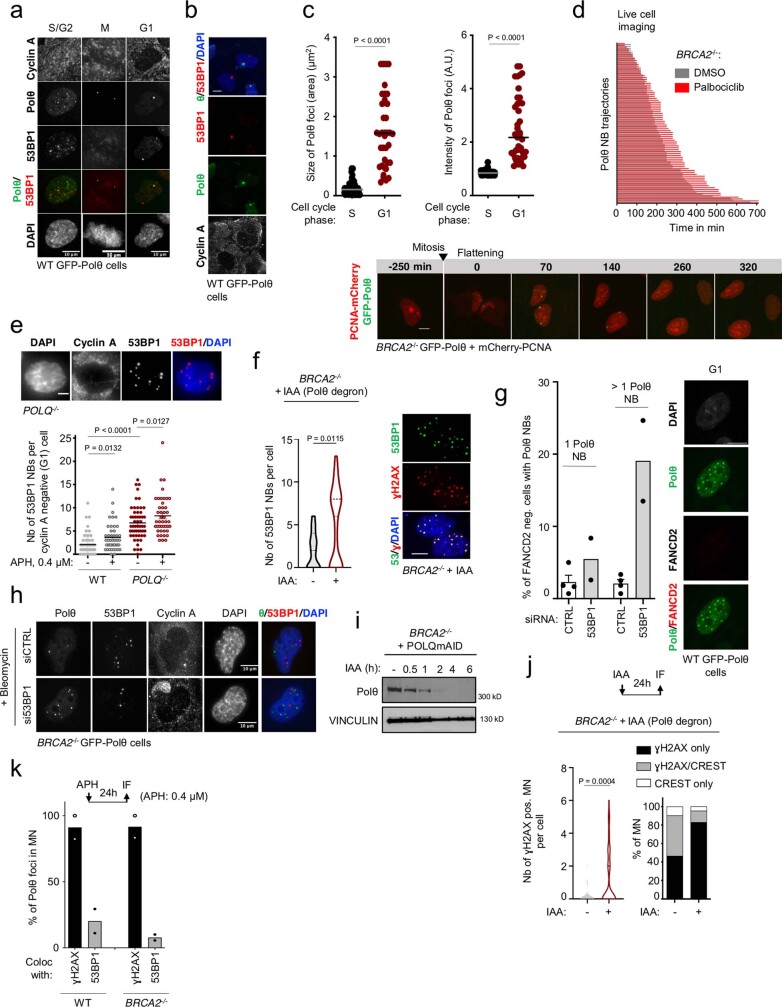
related to Fig. 4. Polθ forms nuclear bodies (NBs) in G1. **a**, **b**, Representative images of Polθ nuclear bodies (Polθ NBs) and 53BP1 nuclear bodies (53BP1 NBs) in cyclin A-negative (G1) cells. **c**, Size and intensity of Polθ foci in indicated cell cycle phases. From left to right, n = 98, 15, 86, 41. **d**, Trajectories of individual Polθ NBs in *BRCA2*^*−/−*^ RPE-1 cells blocked in G1 (Palbociclib treatment). Representative images of Polθ NBs from formation at mitotic exit (flattening) to dissolution in G1 (PCNA-negative cells) *in BRCA2*^*−/−*^ cells. **e**, Representative images and quantification of 53BP1 NBs in WT and *POLQ*^−/−^ cells upon indicated treatment. From left to right, n = 45, 46, 53, 47. **f**, Representative images and quantification of 53BP1 NBs in *BRCA2*^*−/−*^ cells following Polθ depletion (IAA treatment). From left to right, n = 43, 29. **g**, **h**, Representative images and quantification of Polθ NBs in FANCD2-negative (G1) cells upon indicated treatment. **i**, Immunoblot analysis at indicated time points upon Polθ degradation (IAA treatment). In (g), from left to right, n = 561, 448 (4, 2 replicates). **j**, Quantification of micronuclei (MN) positive for γH2AX and/or CREST (centromere marker) in *BRCA2*^*−/−*^ cells following Polθ degradation (IAA treatment). From left to right, n = 75, 57; among two replicates. **k**, Quantification of Polθ foci in micronuclei (MN) in indicated cell lines. Scale bars represent 5 μm, except where indicated. Data represents three biological replicates, except where indicated. Data shows mean +/− S.E.M., except box plots (c) showing median with quartiles. For (c,f,j) two-tailed Mann-Whitney test; for (e) unpaired t-test was performed.

The persistence of under-replicated DNA can result in the formation of 53BP1 nuclear bodies and micronuclei in the next G1 phase^45^. 53BP1 nuclear bodies represent a second opportunity to repair these lesions, in case mitotic DNA synthesis proves insufficient. Similarly to 53BP1, Polθ formed large, replication stress-induced nuclear bodies (Polθ nuclear bodies) and localized to some micronuclei in G1 (Fig. 4a,b and Extended Data Fig. 8a–c). Polθ nuclear bodies were frequently associated with γH2AX, but poorly co-localized with 53BP1 (Fig. 4a and Extended Data Fig. 9a,b). By following each Polθ nuclear body from formation to resolution by time-lapse microscopy, we found that in contrast to 53BP1 nuclear bodies—which are resolved in S phase^46^—Polθ nuclear bodies were resolved in G1 (Extended Data Fig. 9d and Supplementary Video 5). In addition, *POLQ*^*−/−*^ cells exhibited increased levels of 53BP1 nuclear bodies and—conversely—53BP1 knockdown increased Polθ nuclear body formation (Extended Data Fig. 9e–h). Together, our data suggest that mitotic Polθ repair and mitotic DNA synthesis–53BP1 act as parallel pathways to mitigate replication stress and repair unresolved lesions in mitosis and during the next interphase.

## Mitotic DSB repair preserves the genome

**Extended Data Fig. 10:**
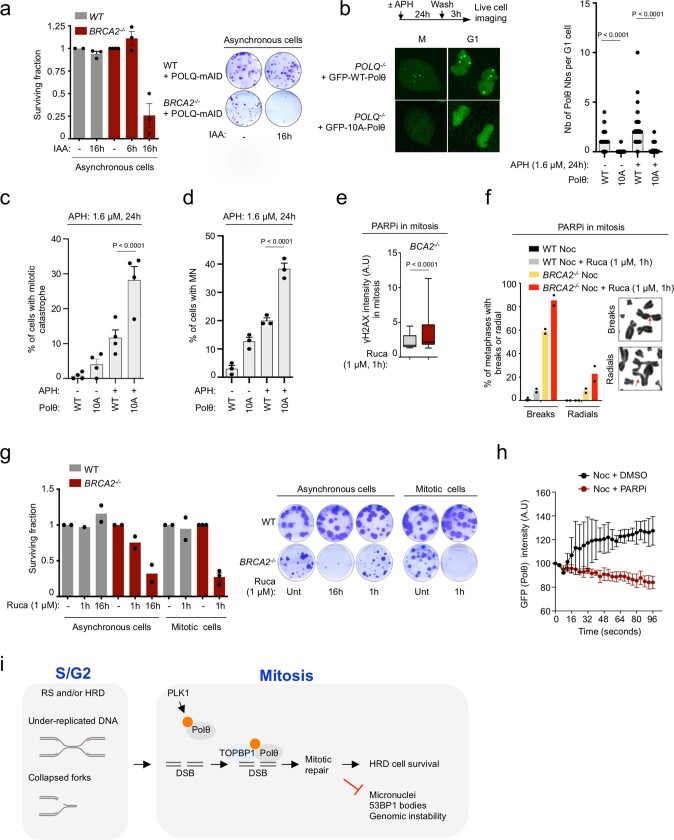
related to Fig. 4. Polθ acts in mitosis to preserve genome integrity and HR-deficient cell survival. **a**, Representative images and quantification of 14-days clonogenic survival upon Polθ depletion in asynchronous cells. **b**,**c**,**d**, Quantification of Polθ NBs (b), mitotic catastrophe (c), and micronuclei (MN) (d) in indicated cell lines upon indicated treatment. In (b–d), data represented belongs to the same experiment, from left to right, n = 75, 52, 86, 65. **e**, Quantification of γH2AX intensity upon PARPi (Rucaparib) treatment in mitosis. From left to right, n = 51, 68; among two replicates. **f**, Representative images and quantification of chromosomal rearrangements (breaks and radials) in WT and *BRCA2*^*−/−*^ cells treated with or without PARPi for 1 h in mitosis. At least 50 metaphase spreads were analyzed in each condition in each replicate (two). **g**, Representative images and quantification of clonogenic survival upon PARPi (Rucaparib) treatment in asynchronous and mitotic cells. Columns without error bars represent one, columns with error bars represent two replicates. **h**, Quantification of Polθ recruitment to laser microirradiated stripes in mitosis. 10 cells were analyzed for both conditions, among two replicates. **i**, Extended model for function of Polθ in mitosis. Scale bars represent 5 μm. Data represents three biological replicates. Data shows mean +/− S.E.M., except box plots (f) showing median with quartiles. For (b–d) Friedman test, corrected with Dunn’s multiple comparisons test, for (e) two-tailed Mann-Whitney test was performed.

Finally, we evaluated the role of Polθ repair in mitosis in maintaining genome integrity and HR-deficient cell survival. To that end, we used the mAID system to deplete Polθ specifically in mitosis in wild-type and *BRCA2*^*−/−*^ cells (Extended Data Fig. 9i). We found that mitotic Polθ depletion led to an increased number of unrepaired mitotic DSBs, as measured by γH2AX foci in mitosis and micronuclei in the next G1 phase (Fig. 4c and Extended Data Fig. 9j,k). We also found that Polθ depletion in mitosis killed *BRCA2*^*−/−*^ cells without affecting the survival of wild-type cells (Fig. 4d and Extended Data Fig. 10a). Together, our data highlight the crucial role of mitotic Polθ repair in maintaining genome integrity and HR-deficient cell survival.

Further strengthening this notion, we found that expression of the Polθ(10A) mutant—which cannot be phosphorylated by PLK1—did not rescue mitotic defects occurring on *POLQ* loss. Whereas the expression of wild-type Polθ rescued mitotic DSB accumulation, micronuclei formation and synthetic lethality with BRCA2 loss, the expression of Polθ(10A) did not rescue any of these phenotypes of *POLQ*^*−/−*^ cells (Fig. 4e–g). When challenged with replication stress, live microscopy revealed that *POLQ*^*−/−*^ cells expressing Polθ(10A) showed an increased frequency of mitotic catastrophe and micronuclei formation, together with an absence of Polθ nuclear bodies (Extended Data Fig. 10b–d and Supplementary Videos 4 and 6). This indicates that PLK1-mediated regulation Polθ in mitosis is essential for its role in maintaining genome integrity.

Finally, we speculated that HR-deficient cells might be extremely vulnerable to any perturbation in the process of mitotic repair. Notably, we found that 1 h of PARP inhibitor (PARPi) treatment in mitotic *BRCA2*^*−/−*^ cells induced the formation of DSBs and chromosomal abnormalities and was sufficient to kill *BRCA2*^*−/−*^ but not wild-type cells, similarly to Polθ depletion (Extended Data Fig. 10e–g). In addition, Polθ recruitment to mitotic DSBs was abolished by PARPi treatment^47^, similar to reports on interphase cells^24,25^ (Extended Data Fig. 10h). Our data suggest that PARPi cytotoxicity in HR-deficient cells is the result, at least partially, of its effect on mitotic DSB repair, possibly through trapping PARP enzymes at sites of mitotic DSBs and/or preventing Polθ recruitment and activity.

In sum, this work demonstrates how phosphorylation by the mitotic kinase PLK1 controls DSB repair activity in mitosis. PLK1 phosphorylation restricts the classical DSB repair pathways HR and NHEJ, in contrast, it enables mitotic end-joining repair by Polθ. In the absence of mitotic Polθ repair, HR-deficient cells experience extreme genomic instability, leading to cell death, providing a possible explanation of the synthetic lethal relationship between Polθ and HR (Fig. 4h and Extended Data Fig. 10i).

Our findings, supported by those of a recent report^48^ highlight the key importance of mitotic DSB repair in maintaining genome integrity. Future research concerning the function and regulation of mitotic repair factors (such as TOBPB1, PARP1 and RHINO) will help not just to further our knowledge, but to exploit therapeutic opportunities.

## Methods

### Cloning and plasmids

All PCRs were performed using the Fidelio polymerase (Ozyme) and cloning was performed using either Gibson assembly mix (NEB or Codex) or T4 DNA ligase (NEB). Exogenous expression of all Polθ constructs (GFP–Polθ) used in this study was achieved by cloning full-length *POLQ* fused with a C-terminal Flag-P2A-BLAST cassette in the PiggyBac CAG eGFP vector (Addgene, #40973) between BsrGI and SacI restriction sites. The P2A-BLAST cassette was amplified from the eFlut P2A-BLAST plasmid (gift from J. Stewart-Ornstein) with forward primer containing the Flag sequence. For degron experiments, the Flag-mAID cassette was amplified from pMK288 (mAID-Bsr, Addgene 72826), fused to P2A-BLAST and cloned in the PiggyBac CAG eGFP-POLQ. TIR1 expression was achieved using the retroviral vector: pBabe Blast osTIR1-9Myc (Addgene 80073). Polθ(10A) and Polθ(4A) were obtained by amplification of DNA fragments with the desired mutations (gBlocks from Integrated DNA Technologies) cloned into PiggyBac CAG eGFP-POLQ-Flag-P2A-BLAST between PflFI and NheI restriction sites. For knock-in experiments, the KI-POLQ-E30-NeonGreen cassette was obtained for *POLQ* knock-in, and the Flag-mAID-Zeocin and the Flag-mAID-Hygromycin cassettes were obtained for *BRCA2* knock-in after assembly of PCR fragments amplified from mNeonGreen plasmid (gift from D. Fachinetti), pMK288 (mAID-Bsr, Addgene 72826) and pMK287 (mAID-Hygro, Addgene 72825) plasmids. PCRs were performed with 600 bp of homologous sequences flanking the gRNA site in the last exons of *POLQ* and *BRCA2* and finally cloned in puc19 vector. pOZ-53BP1^WT^ and pOZ-53BP1^AA^ (containing the T1609A/S1618A mutations) plasmids were gifts from D. Chowdhury. Plasmids allowing bacterial expression of His–TOPBP1 BRCT0-2, GST–TOPBP1 BRCT4-5 and GST–TOPBP1 BRCT7-8 were gifts from J. N. Mark Glover.

### Cell culture and generation of cell lines

We made use of hTERT-RPE-1 (human retinal epithelium), HEK-293T (human embryonic kidney) and HeLa (human cervical adenocarcinoma) cells. All cell lines were obtained from ATCC and were grown in Dulbecco’s modified Eagle’s medium (DMEM) with nutrient Mixture F12 (for RPE-1) or DMEM (for HEK and HeLa) (Gibco) and supplemented with 10% fetal calf serum (FCS), 2 mM glutamine, and 200 IU ml^−1^ penicillin. Cells were incubated at 37 °C in 5% CO_2_. Throughout, wild-type GFP–Polθ refers to the exogenous expression of GFP–Polθ in RPE-1 *TP53*^*−/−*^*;POLQ*^*−/−*^ cells, *BRCA2*^*−/−*^ GFP–Polθ refers to the exogenous expression of GFP–Polθ in RPE-1 *TP53*^*−/−*^*;BRCA2*^*−/−*^*;POLQ*^*−/−*^ cells. When experiments were performed in HeLa or HEK-293T, the cell line origin is specified in the figure. Expression of GFP–Polθ was obtained after co transfection of GFP–Polθ with transposase (System Biosciences, PB210PA-1) according to the manufacturer’s instructions, with blasticidin selection.

All genome-editing experiments were performed using ribonuclear protein (RNP) complexes composed of recombinant *S. pyogenes* Cas9 nuclease, Alt-R CRISPR–Cas9 CRISPR RNA (crRNA) and trans-activating crRNA (tracrRNA) (Integrated DNA technologies 1081058 and 1072532). Knockout cell lines were obtained after transfection with RNP complexes using RNAiMax (ThermoFisher Scientific). RPE-1 *TP53*^*−/−*^ wild-type and isogenic *BRCA2*^*−/−*^ or *POLQ*^*−/−*^ cell lines were described previously^26^.

Knockout cell lines were generated as follows: *BRCA2*^*−/−*^ GFP–Polθ cells were generated by knocking out *BRCA2* in *POLQ*^*−/−*^ cells complemented with GFP–Polθ. To generate *BRCA2*^*−/−*^ cells expressing GFP-Polθ-mAID, GFP-Polθ-mAID was first introduced in *BRCA2*^*−/−*^ cells and then *POLQ* was knocked out. Knockout of the *BRCA2* gene was obtained after transfection of RNP complexes targeting introns flanking exon2 (*BRCA2*-KO1: GGTAAAACTCAGAAGCGC; *BRCA2*-KO2: GCAACACTGTGACGTACT). Knockout of the *POLQ* gene was obtained after transfection of RNP complexes targeting introns flanking exon2 (*POLQ*-KO1: GGAAGGCTTTTAGGTCAGTA; *POLQ*-KO2: GGCAACGGGGGCAGCTCCGC). TIR1 (Addgene 80073) was expressed to allow Polθ degradation upon IAA treatment.

To generate knock-in cell lines, cells were co-transfected with cassettes (Flag-NeonGreen-mAID-BLAST for *POLQ* knock-in, Flag-mAID-Zeocin and Flag-mAID-Hygromycin for *BRCA2* knock-in) and RNP complexes (*POLQ*-KI: GTGAAAATAGGCGCCAGC; *BRCA2*-KI: GGAGAGTTCCCAGGCCAGTA). Next, cells were selected for 2 weeks in appropriate antibiotic and the resistant population was subcloned in 96 well plate. Clones were screened by PCR.

Retroviral particles containing either TIR1, pOZ-53BP1^WT^ or pOZ-53BP1^AA^ plasmids were obtained by transfection of the retroviral vectors with an expression vector for the VSV-G envelope protein (Addgene, #8454) into HEK-293T cells expressing GAG-pol proteins (Phoenix cells) using LTX (ThermoFisher Scientific). Viral particles containing supernatants were collected and filtered before cell transduction.

Cell lines expressing mCherry–PCNA were generated after random integration by transfecting mCherry-PCNA-19-SV40NLS-4 plasmid (Addgene, #55117), antibiotic selection (neomycin) and cell sorting (mCherry) by flow cytometry. Blasticidin (HEK and HeLa: 5 μg ml^−1^, RPE-1: 21 μg ml^−1^), puromycin (HEK: 5 μg ml^−1^, HeLa: 1 μg ml^−1^, RPE-1: 21 μg ml^−1^), zeocin (HeLa: 600 μg ml^−1^), neomycin (RPE-1: 600 μg ml^−1^) and hygromycin (500 μg ml^−1^) were used at indicated concentrations for cell lines selection. Cell lines have been correctly identified by short tandem repeat (STR) analysis (Eurofins) and were tested every month by qPCR for mycoplasma (Eurofins).

### Chemicals, siRNA and antibodies

Nocodazole (100 ng ml^−1^, Sigma), 3-Indoleacetic acid (IAA, 500 μM, Sigma), RO3306 (referred as CDKi, 5 μM, Sigma), Aphidicolin (0.4 μM to 1.6 μM, Abcam), SiR-DNA (250 nM for time-lapse video microscopy and 1 μM for laser micro-irradiation experiments, Spirochrome), ATRi (2.5 μM, Selleckchem, VE-821), ATMi (10 μM, Tocris, Ku-55933), Palbociclib (1 μM, Selleckchem, PD-0332991), Rucaparib (1 μM, Selleckchem), Volasertib (referred as PLK1i throughout, 200 nM, Selleckchem, Bi6727), Bi2536 (200 nM, Selleckchem), MG132 (10 μM, Sigma), Bleomycin (50 μg ml^−1^, Sigma, B7216), and Polθi (Novobiocin, 200 μM, Sigma) were used at the indicated concentrations.

For RNA interference, we used the following targeted sequences targeting *MDC1* (GUCUCCCAGAAGACAGUGAdTdT), *TOPBP1* (ACAAAUACAUGGCUGGUUAdTdT), *BRCA2* (GAAGAAUGCAGGUUUAAUAdTdT), *BRCA1* (CAGCAGUUUAUUACUCACUAAdTdT), *FANCD2* (GGAGAUUGAUGGUCUACUATdT), *53BP1* (CAGGACAGUCUUUCCACGAdTdT) and LacZ (Control, CGUACGCGGAAUACUUCGAdTdT). Transfection of siRNA oligonucleotides (Eurofins) was performed using Lipofectamine RNAiMax (ThermoFisher Scientific) following the manufacturer’s instructions.

For western blotting, antibodies used in this study were purchased from Millipore (BRCA1 OP92; BRCA2 OP95), Sigma (Anti-phospho-Ser/Thr-Pro MPM2 05-368^49^; Anti-pan-phospho-Ser SAB5700550, β-actin A5316), Santa Cruz Biotechnology (PARP1 sc8007; BARD1 sc-74559), Abcam (DNA ligase III ab185815; MDC1 ab11171), Cell Signaling (His 2365), Abclonal (Pan-phospho-Ser/Thr antibody, AP0893) and Bethyl (EXO1 A302-639A, TOPBP1 A300-111, Biotin A150-109A). The Polθ antibody was a gift from J. S. Hoffmann. For immunofluorescence, antibodies were purchased from Institut Curie antibody platform (HA), Immunovision (CREST HCT-0100), Chromotek (mNeonGreen 32F6), Novus Biologicals (FANCD2 NB100-182), Bethyl Laboratories (H2A.X (Ser139) A300-081A; TOPBP1 A300-111), Abcam (MDC1 ab50003; 53BP1 ab21083), Millipore (H2A.X (Ser139) 05-636; phospho-Histone H3 (Ser10) 06-570; 53BP1 MAB3802), and Santa Cruz Biotechnology (cyclin A sc-271682; PCNA sc-56, PLK1 sc-5504). The rabbit phospho-specific antibodies serum against Polθ (pS1493), was generated by Eurogentec using the following phosphopeptide: LLFDpSFSDDYLV.

### X-ray irradiation

Laser micro-irradiation was performed with a two-photon Ti:Saphire laser (Mai Tai DeepSee, Spectra Physics) at 800 nm with 20% power (from 2.1 W) on an inverted laser scanning confocal microscope equipped with spectral detection and a multi-photon laser (LSM880NLO/Mai Tai Laser, Zeiss, Spectra Physics), using a 40× objective NA1.3 oil DICII PL APO (420852-9870). Images were analysed using the ‘plot Z-axis profile’ function of Fiji (version 2.1.0/1.53c). X-ray irradiations were performed using the CIXD Dual Irradiator (RadeXp platform, Institut Curie).

### Cell extracts, immunoprecipitation and Western blotting

All immunoblotting and immunoprecipitation experiments were performed from nuclear extracts. Nuclei were obtained after cells lysis with nuclei extraction buffer (10 mM HEPES pH 7.5, 10 mM KCl, 1.5 mM MgCl_2_, 34 mM sucrose, 10% glycerol, 1 mM dithiothreitol (DTT), 0.1% Triton X-100). Cells lysates were incubated 5 min on ice and centrifuged for 5 min at 1,300*g*. Nuclei (pellet) were lysed in 300 mM RIPA lysis buffer (300 mM NaCl, 50 mM Tris-HCl, pH 7.5, 0.2 mM EDTA, pH 8.0 and 1% NP-40) for 40 min on ice, benzonase (Sigma) was added during the final 10 min of incubation. Extracts were centrifuged for 20 min at 20,000*g* and protein concentration was determined using BCA kit (ThermoFisher Scientific). For immunoprecipitation experiments, 1 mg of protein extract was diluted to 1 mg ml^−1^ in 150 mM NaCl RIPA buffer, incubated with magnetic agarose beads (Chromotek, GFP Trap (GTMA-20), mNeonGreen-Trap (NTMA-20)) for 2 h at 4 °C. Prior to elution, beads were washed twice with 150 mM NaCl RIPA buffer and once with 300 mM NaCl RIPA buffer. All buffers were supplemented with phosphatase and protease inhibitors (PhosSTOP and cOmplete Protease Inhibitor Cocktail, Roche). Finally, lysates were resolved by NuPAGE Tris-acetate gel (4/8%, Invitrogen) and transferred onto nitrocellulose membrane (Bio-Rad) (wet transfer in 10% Tris-Glycine, 20% methanol, 0.05% SDS, 2 h at 40 V). Membranes were then blocked for 1 h in 5% milk or 10% BSA and hybridized with the desired antibody.

### Polθ protein fragment expression and purification

The Polθ protein fragments (E1424–Q1503) and (E1540–S1660) were expressed using a pET-22b expression vector as a fusion protein with a C-terminal GB1 protein tag and 6×His tag. A TEV site was introduced between the Polθ protein fragment and GB1. The Polθ gene fragments were optimized and synthesized by Genscript for high-level expression in bacteria. For NMR studies, the ^15^N-labelled and ^15^N,^13^C-labelled Polθ samples were produced in *E. coli* BL21 (DE3) Star cells grown in M9 minimum medium containing 0.5 g l^−1^ of ^15^NH_4_Cl for the ^15^N-labelled sample or 0.5 g l^−1^ of ^15^NH_4_Cl and 2 g l^−1^ of ^13^C-glucose for the ^15^N/^13^C-labelled sample. Expression was induced with 0.5 mM IPTG at an optical density at 600 nm (OD_600_) of 0.6 and cells were incubated for 3 h at 37 °C. After centrifugation, cell pellets were resuspended in lysis buffer (TBS 1×, EDTA-free Protease Inhibitor Cocktail, Roche) and homogenized by sonication. The lysate was treated with benzonase nuclease and MgCl_2_ (5 mM) for 20 min at room temperature and then centrifuged (15,000*g*) for 20 min at 4 °C. The supernatant was filtered (0.44 µm) and loaded at 2 ml min^−1^ on a HisTrap HP 5 ml column (GE Healthcare) that was equilibrated with buffer A (TBS 1×, 20 mM imidazole). Proteins were eluted at 1 ml min^−1^ using a linear gradient of imidazole (30 min to reach 100% of buffer B: TBS 1×, 500 mM imidazole). Elution fractions of interest were pooled and diluted to 1 mg ml^−1^ with TBS 1× and the GB1-6×His tag was cleaved by the TEV protease (2% w/w) during an overnight dialysis at 4 °C against TBS 1×. Cleaved protein solution was loaded on a HisTrap HP 5 ml (GE Healthcare) column and the Polθ without GB1-6×His fragment was collected in the flow-through. The quality of the purified protein was analysed by SDS–PAGE and the protein concentration was determined using its absorbance at 280 nm.

### NMR spectroscopy

For NMR studies, samples were dialysed against the NMR buffer (25 mM HEPES, 50 mM NaCl, pH 7) and concentrated at 100–150 μM in the case of the ^15^N-labelled samples used to monitor phosphorylation by PLK1, and at 200–300 μM in the case of the ^15^N,^13^C-labelled samples used for the ^1^H, ^15^N, ^13^C NMR chemical shift assignment experiments. The PLK1 sample was provided by the protein production facility of Institut Curie.

To monitor binding to TOPBP1 BRCT7-8, a sample of phosphorylated ^15^N-labelled Polθ (E1424–Q1503) was diluted down to 60–70 µM and dialysed against the NMR buffer. It was further diluted twofold with either the NMR buffer in order to obtain the reference NMR sample with the free peptide, or a solution of purified TOPBP1 BRCT7-8 (Polθ E1424–Q1503:TOPBP1 BRCT7-8 ratio 1:0.5) in this same buffer in order to obtain the NMR sample containing the bound peptide.

The NMR experiments were performed on the Polθ protein fragments (E1424–Q1503) and (E1540–S1660), either ^15^N- or ^15^N,^13^C-labelled, and dissolved in the NMR buffer (with in addition 7% v/v D_2_O and EDTA-free Protease Inhibitor Cocktail 1× (Roche)). Most experiments were recorded on a 700 MHz Bruker ADVANCE NEO spectrometer equipped with a triple resonance cryogenically cooled probe. ^1^HN, ^13^Cα, ^13^Cβ, ^13^CO and ^15^N resonance frequencies of the non-phosphorylated fragments were assigned at 283 K using 2D ^1^H–^15^N SOFAST HMQC, 3D HNCA, 3D HNCACB, 3D CBCA(CO)NH, 3D HNCO, 3D HN(CA)CO and 3D HN(CA)NNH experiments. All 3D experiments were recorded using non uniform sampling (NUS: 40–50%). To monitor phosphorylation by PLK1, either ^15^N- or ^15^N,^13^C-labelled Polθ protein fragments were mixed with PLK1, and a series of 16 2D NMR ^1^H-^15^N SOFAST HMQC experiments were recorded at 298 K during 8 h, each experiment being a 1,536 × 160 dataset acquired with 80 scans during 30 min^50^. The temperature was then set to 283 K and 2D ^1^H–^15^N SOFAST HMQC, 3D HNCA, 3D HNCACB, 3D CBCA(CO)NH and 3D HN(CA)NNH experiments were recorded, in order to assign the resonance frequencies of the phosphorylated fragments (here again with NUS at 40–50%). For Polθ (E1424–Q1503) fragment, resonance assignment was performed at two different stages of the phosphorylation kinetics, first after an incubation with a 1:200 ratio of PLK1, and second after an additional incubation with 1:200 and 1:150 ratios of PLK1. For Polθ (E1540–S1660) fragment, resonance assignment was performed after an incubation with a 1:200 ratio of PLK1.

To identify the TOPBP1 BRCT7-8 binding site in Polθ protein fragment F1, 2D ^1^H–^15^N HSQC experiments were recorded on a ^15^N-labelled Polθ (E1424–Q1503) fragment, either phosphorylated or not, in the absence and presence of TOPBP1 BRCT7-8 at a 1:0.5 ratio, each experiment being a 1,536 × 160 dataset acquired with 800 scans during 5 h. The data were processed using Topspin 3.6 (Bruker) and analysed with CCPNMR Analysis Software^51^. ^1^HN, ^13^Cα, ^13^Cβ, ^13^CΟ and ^15^N resonance assignments of Polθ protein fragments (E1424–Q1503) and (E1540–S1660) were deposited at the Biological Magnetic Resonance Data Bank (BMRB) under the accession numbers 51900 and 51939 for the non-phosphorylated forms and 51920 and 51940 for the phosphorylated forms, respectively.

### Bio-layer interferometry

Biomolecular interactions between the Polθ peptides and TOPBP1 BRCT7-8 were measured using the BLI technology on an Octet RED96 instrument (FortéBio) with streptavidin biosensors. The Polθ peptides (E1472–Y1498) were synthesized by Genecust with an N-terminal biotin. Peptides sequences are (pS being a phosphorylated serine): 4S-P: Biotin-GSG-EGENLPVPETpSLNMpSDpSLLFDpSFSDDY and 4S: Biotin-GSG-EGENLPVPETSLNMSDSLLFDSFSDDY. The biosensors were hydrated for 20 min in the TOPBP1 BRCT7-8 buffer (25 mM HEPES, 50 mM NaCl, 5 mM β-mercaptoethanol). Non-specific interactions between TOPBP1 BRCT7-8 and the biosensors were characterized by performing the binding assays without loading the biotinylated peptides onto the streptavidin biosensors. In these conditions, no interaction was observed. For the binding assays, a concentration of 5 μM of biotinylated peptides was used to attach them on the streptavidin sensors, and TOPBP1 BRCT7-8 was added at different concentrations (62.5–2,000 nM range for 4S and 7.8–500 nM range for 4S-P). 3 cycles of 10-s incubation in the regeneration buffer (1 M NaCl) followed by 10-s incubation in the assay buffer (25 mM HEPES, 50 mM NaCl, 5 mM β-mercaptoethanol, 0.05% Tween) were performed before every kinetics experiment. The *K*_d_ values were measured in the steady-state mode.

### In vitro phosphorylation of full-length Polθ and Polθ E1424–Q1503

Immunoprecipitates of GFP–Polθ (2 h GFP trap at 4 °C, followed by 2 washes with 150 mM NaCl buffer and 1 wash with 300 mM NaCl) or 1.2 μg of RBER-CDC25tide (Reaction Biology, 0590-0000-1) were resuspended in 30 μl kinase buffer (Cell Signaling, 9802) containing 50, 100 or 500 ng of recombinant PLK1 (Reaction Biology, 0183-0000-1) and 5 μM ATP and 5 μCi [γ-^32^P]ATP. After incubation for 30 min at 30 °C, reactions were stopped on ice by the addition of 2× Laemmli buffer and boiling for 5 min at 95 °C. Samples were separated on a 4–8% Tris-acetate gel (or 10% SDS–PAGE for the CDC25 positive control), and phosphorylated ^32^P-Polθ and CDC25 were detected by autoradiography.

Additionally, in vitro phosphorylation assays were performed on the Polθ (E1424 to Q1503) fragment (Genscript) cloned in the pET-28(b+) vector. In brief, 3 μM of Polθ fragment (E1424 to Q1503) or RBER-CDC25tide (a PLK1 substrate used as a positive control, obtained from Reaction Biology, 0590-0000-1. The CDC25 sequence is: ISDELMDA**T**FADQEAK; the threonine phosphorylated by PLK1 is indicated in bold) were incubated with 0.05 μM of PLK1 enzyme (Reaction Biology, 0183-0000-1) in 20 μl of kinase buffer (25 mM Tris-HCl (pH 7.5), 5 mM β-glycerophosphate, 2 mM DTT, 0.1 mM Na_3_VO_4_, 10 mM MgCl_2_) (Abcam) and 0.2 mM ATP for 2 hours at 30 °C. Reactions were loaded on an acrylamide gel, in which phosphorylated proteins were detected using the Pro-Q Diamond Phosphoprotein Blot Stain Kit (ThermoFisher Scientific, P33356), and total proteins using SYPRO Ruby Protein Gel Stain (ThermoFisher Scientific, S12000) following the manufacturer’s instructions and revealed using ChemiDoc imaging system (Bio-Rad).

### Expression and purification of TOPBP1 BRCT fragments

The TOPBP1 fragment (1–290), also known as TOPBP1 BRCT0-2, was cloned into a pET-22b plasmid (Genscript). The TOPBP1 fragments (559–746) and (1264–1493), also known as TOPBP1 BRCT4-5 and TOPBP1 BRCT7-8, were expressed using pGEX-6P-1 plasmids provided by J. N. Mark Glover^52,53^. The BRCT0-2 fragment contains an 8-his N-terminal tag, the BRCT4-5 and BRCT7-8 a GST tag. All BRCT domains contain a TEV cleaving site. Plasmids coding for TOPBP1 BRCT domains 0-2, 4-5, and 7-8 were transfected into *E. coli* BL21 (DE3) Star cells (Thermo Scientific, EC0114) grown at 37 °C up to an OD_600_ of 0.7, induced with 0.2 mM isopropyl-β-d-thiogalactopyranoside (IPTG) and incubated at 22 °C overnight (16 h). Cell cultures were harvested by centrifugation and resuspended in 25 mM Tris-HCl pH 7.5, 150 mM NaCl, 5 mM DTT and 10% glycerol. The proteins were further purified as described^52,53^. Expression of TOPBP1 BRCT domains was confirmed by immunoblotting using His (BRCT0-2) or GST (BRCT4-5 and BRCT7-8) antibodies.

### Peptide pull-downs

Biotinylated Polθ peptides containing desired phosphorylated serines were purchased from Genecust (4S and 4S-P; S1628/S1635 and S1628-P/S1635-P) and were resuspended in water at concentrations ranging from 0.5 to 2 mg ml^−1^ in accordance with the manufacturer’s instructions. Peptides were coupled to streptavidin beads in 150 mM NaCl on a rotating wheel at room temperature, for 1 h. Beads were washed twice in 150 mM NaCl prior to use in pull-down assays. Nuclear extracts from HeLa cells (350 μg) or 2 ml of bacteria protein extracts were incubated with 1 nmol of desired streptavidin beads-coupled peptide, on a rotating wheel, for 2 h, at room temperature. After incubation, beads were washed three times using 150 mM NaCl RIPA buffer, and twice using 300 mM NaCl before elution in 2× Laemmli buffer. All buffers used were supplemented with phosphatase and protease inhibitors. Eluted proteins were analysed by SDS–PAGE to identify peptide partners. The same amount of peptides used for pull-downs was loaded into a separate gel, and biotin signal was detected in the migration front. Peptide sequences are (pS being a phosphorylated serine): 4S-P (1482/86/88/93): biotin-GSG-EGENLPVPETpSLNMpSDpSLLFDpSFSDDY. 4S (1482/86/88/93): biotin-GSG-EGENLPVPETSLNMSDSLLFDSFSDDYS. 1628-P/S1635-P: biotin-GSGTRQNHpSFIWSGApSFDLSPGLQRILDKVSS. S1628/S1635: biotin-GSGTRQNHSFIWSGASFDLSPGLQRILDKVS.

### Mass spectrometry analyses

For the identification of Polθ binding partners by mass spectrometry, Polθ immunoprecipitation was performed as described above in 5 mg of nuclear extract of HEK-293T NeonGreen-POLQ knock-in cells. Lysates were incubated with mNeonGreen-Trap Magnetic Agarose beads (Chromotek) for 2 h at 4 °C and beads were washed twice with 150 mM NaCl buffer, once with 300 mM NaCl and three times in 25 mM ammonium bicarbonate. Finally, beads were resuspended in 100 µl 25 mM ammonium bicarbonate and digested by adding 0.2 μg of trypsin/LysC (Promega) for 1 h at 37 °C. Samples were then loaded into custom-made C18 StageTips packed by stacking one AttractSPE disk (SPE-Disks-Bio-C18-100.47.20 Affinisep) and 2 mg beads (186004521 SepPak C18 Cartridge Waters) into a 200 µl micropipette tip for desalting. Peptides were eluted using a ratio of 40:60 MeCN:H_2_O + 0.1% formic acid and vacuum concentrated to dryness. Peptides were reconstituted in injection buffer (0.3% TFA) before liquid chromatography–tandem mass spectrometry (LC–MS/MS) analysis by using an RSLCnano system (Ultimate 3000, Thermo Scientific) coupled to an Orbitrap Exploris 480 mass spectrometer (Thermo Scientific) as described previously^54^. Mass spectrometry analysis was performed on three independent experiments. NeonGreen-Trap Magnetic Agarose beads incubated in HEK native cell extracts were used as control. For Polθ phosphorylated sites identification by mass spectrometry, Hela cells expressing GFP–Polθ were blocked 16 h in nocodazole. DMSO or Volasertib was added during the last hour of nocodazole treatment. 5 mg of nuclear extract were incubated with GFP Trap Magnetic Agarose beads (Chromotek) for 2 h at 4 °C and beads were washed once with 150 mM NaCl buffer, twice with 300 mM NaCl and twice with 500 mM NaCl buffer. Finally, beads were resuspended in 100 µl 25 mM Ammonium bicarbonate and digested by adding 0.2 μg of trypsin/LysC (Promega) for 1 h at 37 °C. Samples were then desalted and peptides were reconstituted in injection buffer before LC–MS/MS analysis as described previously in the Polθ binding partners identification. Mass spectrometry analysis was performed on five independent experiments. GFP Trap Magnetic Agarose beads incubated in HeLa native cell extracts were used as controls. For Polθ peptide (4S and 4S-P) binding partners identification by mass spectrometry, peptide pull-downs were performed in 666 μg of nuclear extract of HeLa cells. Lysates were incubated with streptavidin beads-coupled peptide, on a rotating wheel for 2 h at room temperature. After incubation, beads were washed three times using 150 mM NaCl buffer and twice using 300 mM NaCl. Beads were resuspended, digested and desalted, and then peptides were reconstituted in injections buffer before LC–MS/MS analysis, as described for Polθ binding partners identification and by using an Orbitrap Eclipse mass spectrometer. Mass spectrometry analysis was performed on five independent experiments. Pull-downs of streptavidin beads incubated in nuclear extract of HeLa cells were used as control.

The mass spectrometry proteomics data have been deposited to the ProteomeXchange Consortium (http://proteomecentral.proteomexchange.org) via the PRIDE^55^ partner repository with the dataset identifier PXD029585 (username: reviewer_pxd029585@ebi.ac.uk, password: Tv3OwInu) for Polθ binding partners and project accession: PXD036486 (username: reviewer_pxd036486@ebi.ac.uk, password: GqeYlT33) for Polθ phosphorylated sites quantification and project accession: PXD042167 (Username: reviewer_pxd042167@ebi.ac.uk, password: JdyDITGR) for Polθ peptide 4S-P binding partners identification.

### Mitotic DSB repair assays

#### For mitotic DSB induced by RNP–Cas9

Cells were plated in 4× 10-cm dishes per condition and synchronized by overnight treatment with nocodazole. Next, mitotic cells were collected by shake off, centrifuged and counted. 250,000 mitotic cells were plated in 1 ml of nocodazole containing medium and transfected with an RNP complex targeting one of two AAVS1 loci located on chromosome 19 (AAVS1 gRNA locus 1: TGTGGCCTGGGTCACCTCTA; AAVS1 gRNA locus 2: GGGGCCACTAGGGACAGGAT). A total of 7 pmol of Cas9 was incubated at room temperature with 8.4 pmol of annealed crRNA–tracrRNA in a total volume of 100 µl Opti-MEM for 10 min. RNAimax (4 µl) was then added to the mix, and cells were transfected after 5 min of incubation. Seven hours post transfection, cells were pelleted, and genomic DNA extraction was performed using Nucleospin tissue kit (Macherey-Nagel). A fraction of mitotic cells was kept for phospho-histone H3 (Ser10) immunostaining. PCR around the break was performed using Terra polymerase (Takara) (30 cycles: 95 °C 10 s, 59 °C 20 s, 68 °C 20 s) (locus 1: For2bp GAGGACATCACGTGGTGCAG, Rev2bp CTGCCGTCTCTCTCCTGAGT; locus 2: For8bp GGTGTGTCACCAGATAAGGAATCTGC, Rev8bp GAGCTGGGACCACCTTATATTCC). PCR products were digested for 2 h with HphI (25 U final)(NEB), purified, using Nucleospin PCR cleanup kit (Macherey-Nagel), ligated into pCR4 vector, (4 µl of PCR per ligation) (TOPO-TA cloning kit, ThermoFisher Scientific), and subsequently transformed into NEB5 bacteria (ThermoFisher Scientific). Colonies were subjected to Sanger sequencing using T7 or T3 primer (Eurofins). Repair products missing one or both primer sequences and/or retaining HphI cut site were excluded from analysis. Data were plotted in R using ggplot2.

#### Extrachromosomal substrate to monitor mitotic end-joining repair

##### Construction of a DNA repair substrate

To assess mitotic end-joining repair, a DNA repair substrate was designed to mimic resected DNA ends containing four microhomologies, as previously described^29^. The repair substrate was built by ligating a PCR product (GFP sequence) to DNA oligonucleotide duplexes on both sides. The PCR product (747 bp) was amplified from a GFP sequence (PCAG vector, Addgene) using the following oligonucleotide sequences (Fwd core GFP: CAAGTGGTCTCAGACTGTGAGCAAGGGCGAGGAGCTG, Rev core GFP: GCCGAGGTCTCCGTCAGCTTGTACAGCTCGTCCATGCCGAG), digested by the BsaI-HF v2 enzyme (NEB) to reveal 4 nucleotides (nts) complementary to the DNA oligonucleotide duplexes and finally, purified (Gel and PCR cleanup kit Macherey-Nagel). In parallel, two oligonucleotide duplexes (1 and 2) with ~70 nts overhangs were made after annealing (95 °C 10 min, 25 °C 30 min) of the following oligonucleotides. Oligonucleotides for duplex 1 (17 nt dsDNA, 69 nt ssDNA): oligo 1-A, CATCGCTTAGCTGTATA; oligo 1-B, TGACTATACAGCTAAGCGATGCTCTCACCGAGCGTATCTGCTGGG TTGTGGATGAATTACATATGCTGGGAGAACCAAGATTGG**GCAG**TT. Oligonucleotides for duplex 2 (15 nt dsDNA, 71 nt ssDNA): oligo 2-A, CTCACACCCATCTCA; oligo 2-B: AGTCTGAGATGGGTGTGAGAGTGAAGATCCTCACCTTCGGAGTACTCCTTCTTTTGACCATTGATACGATACTTCTCAGCCGAG**CTGC**TT.

Oligonucleotide duplex 1 and duplex 2 (100 pmol of each) were ligated to the core GFP PCR (10 pmol) using T7 ligase (NEB). The resulting duplex 1–GFP–duplex 2 DNA fragment was then phosphorylated (polynucleotide kinase, NEB) and purified (Nucleospin PCR Cleanup, Macherey-Nagel). In cells, two duplex 1–GFP–duplex 2 DNA fragments could anneal through sequence microhomology (four nucleotides in bold in oligonucleotide sequences) forming DNA end-joining repair product.

##### DNA repair substrate transfection and measurement of end-joining repair efficiency

Cells (interphase or mitotic) were transfected with 150 ng of duplex 1–GFP–duplex 2 substrate using RNAimax reagent and incubated for 1.5 h. Cells were then collected, centrifuged, and resuspended in 2 ml benzonase buffer (50 mM Tris-HCl, 1 mM MgCl_2_, 25 U benzonase), with the addition of nocodazole for mitotic cells, and incubated for 30 min at 37 °C to remove extracellular DNA. DNA was purified using the NucleoSpin tissue kit (Macherey-Nagel).

To measure DNA repair efficiency, a qPCR was performed both on the GFP sequence (to control for the DNA repair substrate transfection) as well as on potential end-joining repair products. Mitotic end-joining repair efficiency was calculated using ΔΔ*C*_t_ between GFP qPCR and end-joining qPCR using SYBR Green Master Mix (ThermoFisher Scientific).

Primers used for qPCR. GFP: For qPCR core, CTCACACCCATCTCAGACTGTGAGCAA; Rev qPCR GFP, CAGCTTGCCGTAGGTGGCATCG. End joining: For qPCR EJ, GGGTTGTGGATGAATTACATATGCTGG; Rev qPCR EJ, CGGAGTACTCCTTCTTTTGACCATTGATAC.

All oligonucleotides and primers were ordered from Eurofins.

### Immunofluorescence microscopy

Cells grown on coverslips were fixed for 15 min in 2% paraformaldehyde, permeabilized for 10 min in 0.5% Triton X-100, blocked for 30 min in BSA 5%, 0.1% Triton X-100, immunostained for 1 h with primary antibodies, 45 min with secondary antibodies (Highly Cross-Adsorbed Secondary Antibodies Alexa Fluor, ThermoFisher Scientific) and mounted Vectashield mounting medium containing DAPI (Vector Laboratories). The same protocol was applied to mitotic cells, which were collected by shake off and centrifuged onto glass slides using a cytospin centrifuge (400*g*, 5 min) before fixation. For staining with PCNA or TOPBP1 antibodies, cells were fixed with ice-cold methanol for 20 min on ice before blocking and immunostaining. Biotin azide-EdU click-it reaction was performed after fixation and permeabilization by incubating cells with a mix of Picolyl biotin, CuSO_4_, THPTA (Jena Bioscience) and sodium ascorbate in PBS for 30 min in the dark followed by an incubation with streptavidin conjugated Alex fluor 647 antibody (ThermoFisher Scientific) during 1 h at room temperature. Images were taken at 60× or 100× magnification using Upright Spinning disk Confocal Microscope (Roper/Zeiss) or at 40× magnification using DeltaVision (Deconvolution) Image Restoration Microscope. All images were taken with a *Z*-stack of 0.4 μM on 8 μM range around focus and projected using maximum intensity projection. Images were analysed using the Fiji software^56^. All scale bars represent 5 μm unless stated otherwise.

### Automatic microscopy for screening Polθ foci formation

The Human DDR siRNA library comprising 1,746 single siRNAs representing 582 human genes (3 siRNAs per gene) was obtained from ThermoFisher Scientific (A30089). For controls, siRNAs targeting GL2, kinesin family member 11 (*KIF11*) and *GM130* were used to assess transfection efficacy and induced cell toxicity. *FANCD2* siRNA (GGAGAUUGAUGGUCUACUATdT, defined as FANCD2-ctrl in Supplementary Table 1) was used as a positive control inhibiting Polθ foci formation. RPE-1 cells expressing GFP–Polθ (250 cells per well in 384-well plates) were grown overnight. siRNAs were incubated 15 min with 0.05 μl Interferin reagent (Polyplus transfection) and Opti-MEM (ThermoFisher Scientific) prior to being transferred into each 384-well plate wells, to obtain a 10 nM final concentration. Cells were irradiated 72 h after transfection, at a dose of 5 Gy (226 s) with the CIXD irradiator (RadeXp platform, Institut Curie) and incubated at 37 °C, 5% CO_2_ for 2 h. Cells were fixed in 3% paraformaldehyde solution (Sigma-Aldrich, HT5014) for 15 min with MAP-C (Titertek), washed once in 1× PBS and quenched in 50 mM NH_4_Cl solution for 10 min. Cells were then blocked with 1% BSA solution for 15 min and permeabilized with 0.5% Triton X-100 for 5 min. Nuclei were stained with 0.2 μg ml^−1^ Hoechst 33342 (DNA marker,1:500, Sigma, 14533). Image acquisition was performed using the INCell analyser 6500 HS automated system (GE Healthcare) at a 40× magnification (Nikon 40×/0.95, Plan Apo), using the same exposure time for all plates in the experiment and across replicate experiments. Plates were loaded onto the INCell system with Kinedx robotic arm (PAA). 16-bit images of six fields in each well were acquired, each containing channels for Hoechst 33242 (DAPI, wave 1) and intrinsic GFP–Polθ (FITC, wave 2). Computational image processing operations were performed using INCell Analyser 3.7 Workstation software (GE Healthcare). Polθ was then identified as organelles in the nuclei area and quantified by the average intensity of pixels within the defined organelles region. A binary threshold was applied to discriminate cells containing at least of 5 Polθ foci resulting in “% cells multi-foci” (Supplementary Table 1). For each feature or phenotypic class depicted from the applied cell threshold described, plate positional effects were corrected using Tukey’s two-way median polishing^57,58^, which iteratively subtracts row, column and well median computed across all plates within a replicate. Corrected values were normalized as follows: sample median and median absolute deviation (MAD) were calculated from the Interferin-treated population of each internal plate data points (named as Ref pop) and used to compute robust Z-scores^59^ (RZ scores), according to the formula: RZ score = (compound value – median (reference population))/(1.14826  × MAD (reference population)). Where the compound value corresponds to the treated well and MAD is defined as the median of the absolute deviation from the median of the Interferin-treated population. A gene was identified as a hit if |RZ score| >2 points in the same direction for at least 2 siRNAs targeting the same gene in at least 2 replicate experiments. The screen was done in three biological replicates. For each siRNA, the median RZ score of each of the three replicate experiments is shown in Fig. 1a.

### Time-lapse video microscopy

To determine Polθ foci formation during cell cycle progression, cells expressing GFP–Polθ and PCNA-mCherry were plated on 35 mm Ibidi μ-Dishes (Ibidi, 81156) and imaged every 10 min for 16 h on an Inverted Eclipse Ti-E (Nikon) microscope equipped with a stage-top incubator and CO_2_ delivery system, 40× (1.4 NA) oil objective, a Spinning disk Yokogawa CSU-X1 unit integrated in Metamorph software by Gataca Systems equipped with Camera EMCCD. Images were mounted in SoftWoRx and analysed using the Fiji software (1.53c). Aphidicolin (1.6 μM unless stated otherwise) was added to the cells for 24 h and removed 3 h before acquisition. For nuclear bodies resolution, palbociclib was added 30 min before starting acquisition.

To determine mitotic timing by phase-contrast video microscopy, 250,000 cells were seeded in 35 mm Ibidi μ-Dishes (Ibidi, 81156) 24 h before imaging. Cells were arrested in RO3306 for 4 h with or without IAA (Polθ depletion) and released in normal growth media before imaging. Cells were immediately imaged every 5 min for 16 h, at 20× using an inverted video microscope (Deltavision) equipped with CoolSNAP HQ2 camera controlled by Softworx software. Cell rounding (mitotic entry) and flattening (mitotic exit) were used as landmarks to calculate mitotic timing. In addition, mitotic timing was also measured from condensation to anaphase. In that case, DNA was stained 30 min prior acquisition by incubating cells with SiR-DNA (250 nM, Spirochrome) and acquisition was performed an Inverted Eclipse Ti-E (Nikon) microscope equipped with a stage-top incubator and CO_2_ delivery system, 40× (1.4 NA) oil objective, equipped with Intensilight Lamp Hg 130W (Nikon) and Camera CoolSNAP HQ (Photometrics). All images were mounted in SoftWoRx and analysed using the Fiji software (1.53c).

### Clonogenic survival assays

For mitotic cells: cells were blocked in G2 using RO3306 (5 μM), released, then blocked in mitosis by nocodazole and subsequently seeded for clonogenic survival. Polθ-mAID cells were blocked 4 h in RO3306 (5 μM) with or without IAA (500 μM) and released in nocodazole with or without IAA for 1 h. In the case of PARPi treatment, cells were blocked 16 h in RO3306 (5 μM) and released in nocodazole with or without Rucaparib (1 μM) for 1 h. After shake off, mitotic cells were washed twice with warm media, counted and seeded for clonogenic survival. For interphase cells: growing cells were treated with IAA (500 μM), or Rucaparib (1 μM) for indicated time, washed twice with warm media and counted. For clonogenic assay upon siRNA transfection, 150,000 cells were plated per well of a 6-well plate and transfected the same day with indicated siRNA. The day after, cells were washed twice, counted and seeded for clonogenic survival. For each condition, two concentrations of cells (*BRCA2*^*−/−*^ cells: 500 and 2000 cells; wild-type cells: 50 and 150 cells) were seeded per well of a 6-well plate. Finally, 10 to 14 days after seeding, colonies were stained with blue methylene (Sigma, 1808) for 2 h at room temperature, washed in water, air-dried overnight and scored manually.

### Metaphase spreading

Cells were blocked in G2 with RO3306 (5 μM) for 16 h, released, then blocked in mitosis by nocodazole and subsequently treated with Rucaparib for 1 h. After shake off, mitotic cells were centrifuged and subsequently resuspended in a pre-warmed hypotonic solution (0.075 M KCl) and maintained at 37 °C for 15 min. Cells were then centrifuged, and pellets were fixed in fresh methanol and stored at −20 °C. After metaphase spreading, air-dried slides were stained with a Gurr/Giemsa/Acetone solution (Gibco, 10582013; Giemsa (KaryoMAX Giemsa stain solution, Gibco, 10092013)) for 5 min and then rinsed twice with Gurr buffer. Slides were air-dried and neo-mounted with anhydrous mounting medium (Merck, 109016). Images were acquired at 100× magnification using DeltaVision (Deconvolution) Image Restoration Microscope.

### AlphaFold calculations

A series of five models were calculated for the complex between TOPBP1 BRCT7-8 and the Polθ peptide E1472–Y1498 in its non-phosphorylated and phosphomimetic (phosphorylated serines being replaced by glutamic acids) states. All models gave a similar representation of the interface, characterized by an iPTM score of 0.36–0.51 and 0.48-0.61 in the case of the non-phosphorylated and phosphomimetic peptides, respectively. The weak effect of phosphomimetic mutations on the AlphaFold calculations is consistent with the poor sensitivity of AlphaFold to single mutations.

### Statistical analysis

Data are shown as mean ± s.e.m. except in violin plots, which show median with quartiles, and box plots, which show median with minimum and maximum values. For all statistically significant comparisons *P* values are displayed on figures and statistical analyses are specidfied in the figure legends. Sample sizes and number of biological replicates are specified in the figure legends. Blinding and randomization were not employed in this study. All statistical analyses were performed with GraphPad Prism 9, except chi-square tests, which were performed using Chi-Square Test Calculator (https://www.socscistatistics.com/tests/chisquare2/default2.aspx).

### Reporting summary

Further information on research design is available in the Nature Portfolio Reporting Summary linked to this article.

## Online content

Any methods, additional references, Nature Portfolio reporting summaries, source data, extended data, supplementary information, acknowledgements, peer review information; details of author contributions and competing interests; and statements of data and code availability are available at 10.1038/s41586-023-06506-6.

### Supplementary information

Supplementary Data This file contains source data for Figs. 2 and 3 and Extended Data Figs. 1, 4, 6, 7 and 9. In raw gel data, molecular weight markers and the region used in the figure are indicated.

Reporting Summary

Supplementary Table 1 List of positive hits from the siRNA screen for Polθ foci formation. Hits with a |RZ score| >2 are shown. Final values in the hit table (Table 1) correspond to the median RZ score of the three siRNAs.

Supplementary Table 2 List of Polθ binding partners identified by mass spectrometry. Endogenous NG-tagged Polθ was immunoprecipitated in nuclear extracts of HEK-293T cells.

Supplementary Table 3 Example of mitotic DNA repair events at indicated locus in wild-type and *POLQ*^*−/−*^ cells. Sequences are shown for both loci.

Supplementary Table 4 List of PLK1 phosphorylation sites identified on Polθ by mass spectrometry. The analysis was performed upon Polθ immunoprecipitation from mitotic HeLa cells were treated with or without PLK1i.

Supplementary Table 5 List recording all PLK1 phosphorylation sites on Polθ identified in this study. Sites identified in silico, by NMR and mass spectrometry are indicated.

Supplementary Table 6 List of binding partners to phosphorylated (4S-P) Polθ peptide identified by mass spectrometry. Polθ pepides (4S-P and 4S) were immunoprecipitated in nuclear extracts of HeLa cells.

Supplementary Video 1 Polθ foci formation (green) in S phase in wild-type cells treated with aphidicolin. Cell cycle phases are marked by changing PCNA patterns (red).

Supplementary Video 2 Polθ foci formation (green) throughout the cell cycle (G2-M-G1) in wild-type cells treated with aphidicolin. Cell cycle phases are marked by changing PCNA patterns (red).

Supplementary Video 3 Polθ foci formation (green) throughout the cell cycle in *BRCA2*^*−/−*^ cells. Cell cycle phases are marked by changing PCNA patterns (red).

Supplementary Video 4 Polθ foci formation (green) throughout the cell cycle in *POLQ*^*−/−*^ cells expressing 10A-Polθ and treated with aphidicolin. Cell cycle phases are marked by changing PCNA patterns (red). The red arrow indicates the cell to follow, the white arrow, the formation of a micronucleus.

Supplementary Video 5 Polθ nuclear bodies (NBs, green) resolution in *BRCA2*^*-/-*^ cells blocked in G1. *BRCA2*^*−/−*^ cells were blocked in G1 by a Palbociclib treatment.

Supplementary Video 6 Polθ foci formation (green) throughout the cell cycle in *POLQ*^*−/−*^ cells expressing wild-type Polθ and treated with aphidicolin. Cell cycle phases are marked by changing PCNA patterns (red).

## Data Availability

The NMR chemical shift data are publicly available: ^1^HN, ^13^Ca, ^13^Cb, ^13^CO and ^15^N resonance assignments of Polθ fragments (E1424–Q1503) and (E1540–S1660) were deposited at the Biological Magnetic Resonance Data Bank (BMRB) under the accession numbers 51900 and 51939 for the non-phosphorylated forms and 51920 and 51940 for the phosphorylated forms, respectively. Mass spectrometry proteomics data have been deposited to the ProteomeXchange Consortium under the identifiers PXD029585, PXD036486 and PXD042167.
