# Publisher Correction: Polθ is phosphorylated by PLK1 to repair double-strand breaks in mitosis

**DOI:** 10.1038/s41586-024-07025-8

**Published:** 2024-01-25

**Authors:** Camille Gelot, Marton Tibor Kovacs, Simona Miron, Emilie Mylne, Alexis Haan, Liza Boeffard-Dosierre, Rania Ghouil, Tatiana Popova, Florent Dingli, Damarys Loew, Josée Guirouilh-Barbat, Elaine Del Nery, Sophie Zinn-Justin, Raphael Ceccaldi

**Affiliations:** 1 INSERM U830, PSL Research University, Institut Curie, Paris, France; 2 Institute for Integrative Biology of the Cell (I2BC), CEA, CNRS, Paris-Saclay University, Gif-sur-Yvette, France; 3 INSERM U830, DNA Repair and Uveal Melanoma (D.R.U.M.), Equipe labellisée par la Ligue Nationale Contre le Cancer, PSL Research University, Institut Curie, Paris, France; 4 CurieCoreTech Mass Spectrometry Proteomics, Institut Curie, PSL Research University, Paris, France; 5 Université de Paris, INSERM U1016, UMR 8104 CNRS, Equipe Labellisée Ligue Nationale Contre le Cancer, Institut Cochin, Paris, France; 6 Department of Translational Research-Biophenics High-Content Screening Laboratory, Cell and Tissue Imaging Facility (PICT-IBiSA), PSL Research University, Institut Curie, Paris, France

**Keywords:** Double-strand DNA breaks, DNA synthesis, Breast cancer

Correction to: *Nature* 10.1038/s41586-023-06506-6 Published online 6 September 2023

In the version of the article initially published, in the right panel of Fig. 2g, the text now reading “4S Polθ” previously read “4S-P Polθ”. The error has been corrected in the HTML and PDF versions of the article.

