# Supplementary material for: Polθ is phosphorylated by PLK1 to repair double-strand breaks in mitosis
Source: Nature. 2023 Sep 6;621(7978):415–22. doi: 10.1038/s41586-023-06506-6 (PMC10499603; doi:10.1038/s41586-023-06506-6)

nature portfolio

https://doi.org/10.1038/s41586-023-06506-6

Supplementary information

Polθ is phosphorylated by PLK1 to repair double-strand breaks in mitosis

In the format provided by the authors and unedited

Source data for Fig 2a. Dotted rectangle correspond to samples included in the manuscript

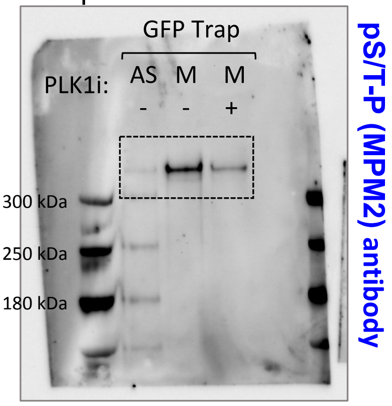

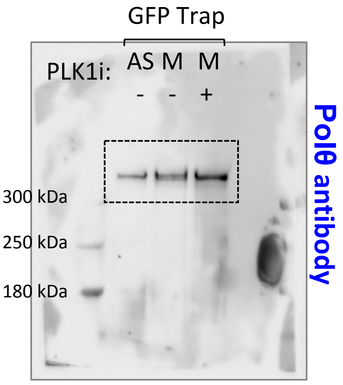

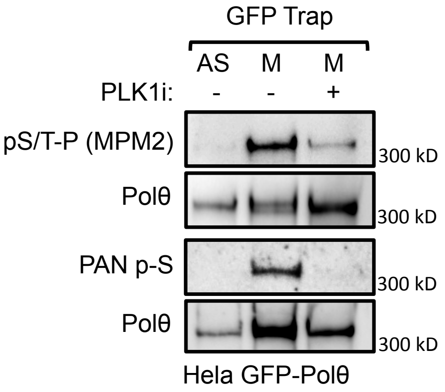

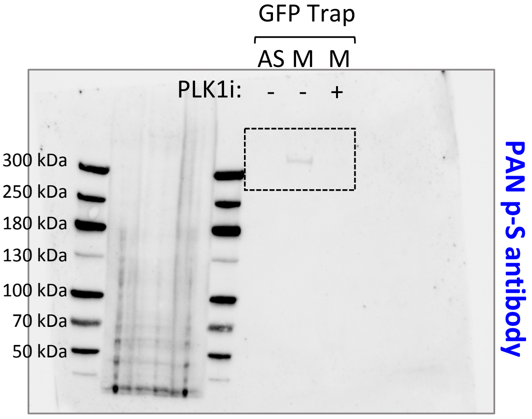

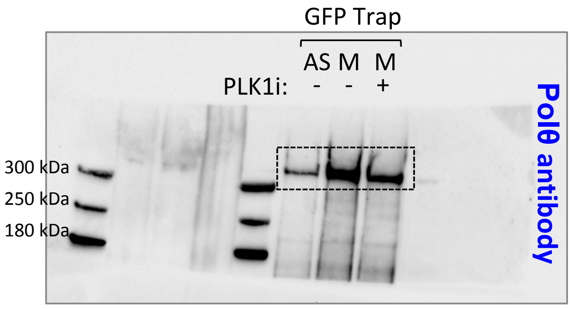

Source data for Fig 2f. Dotted rectangle correspond to samples included in the manuscript

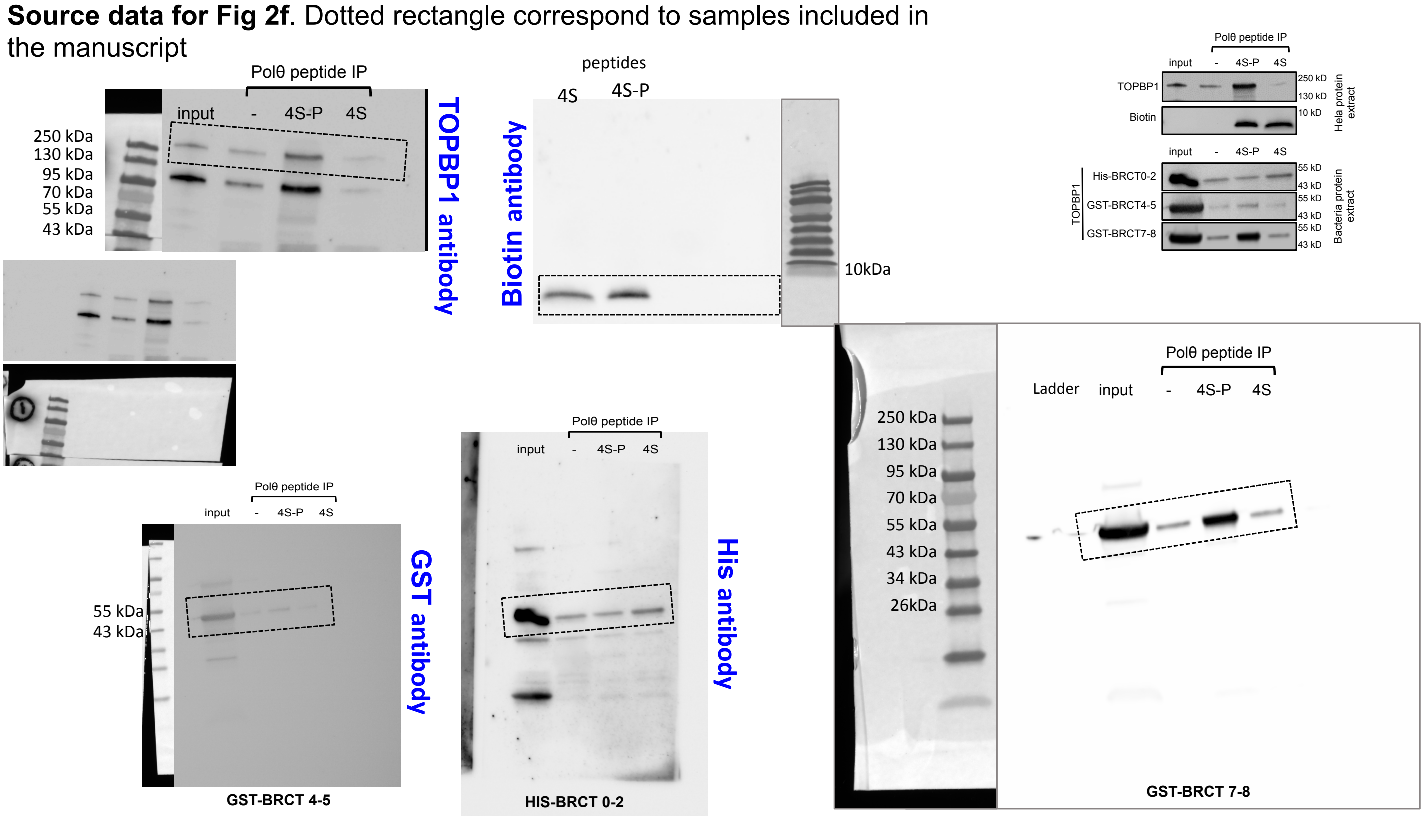

Fig 3e. Dotted rectangle correspond to samples included in the manuscript

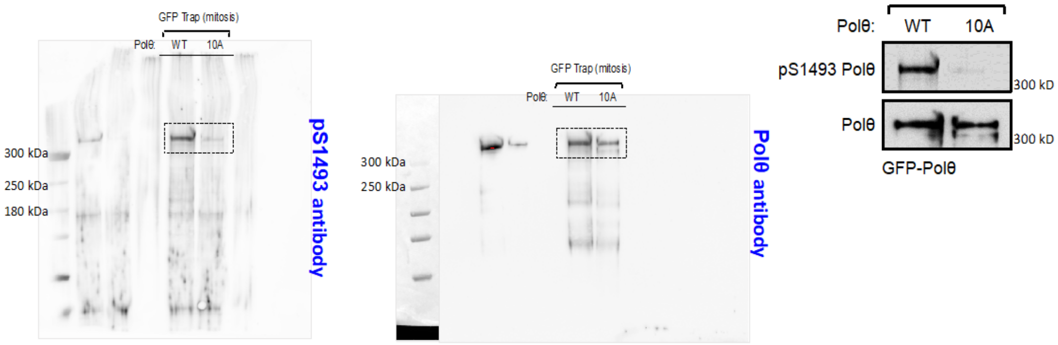

Extended Data Figure 1h. Dotted rectangle correspond to samples included in the manuscript

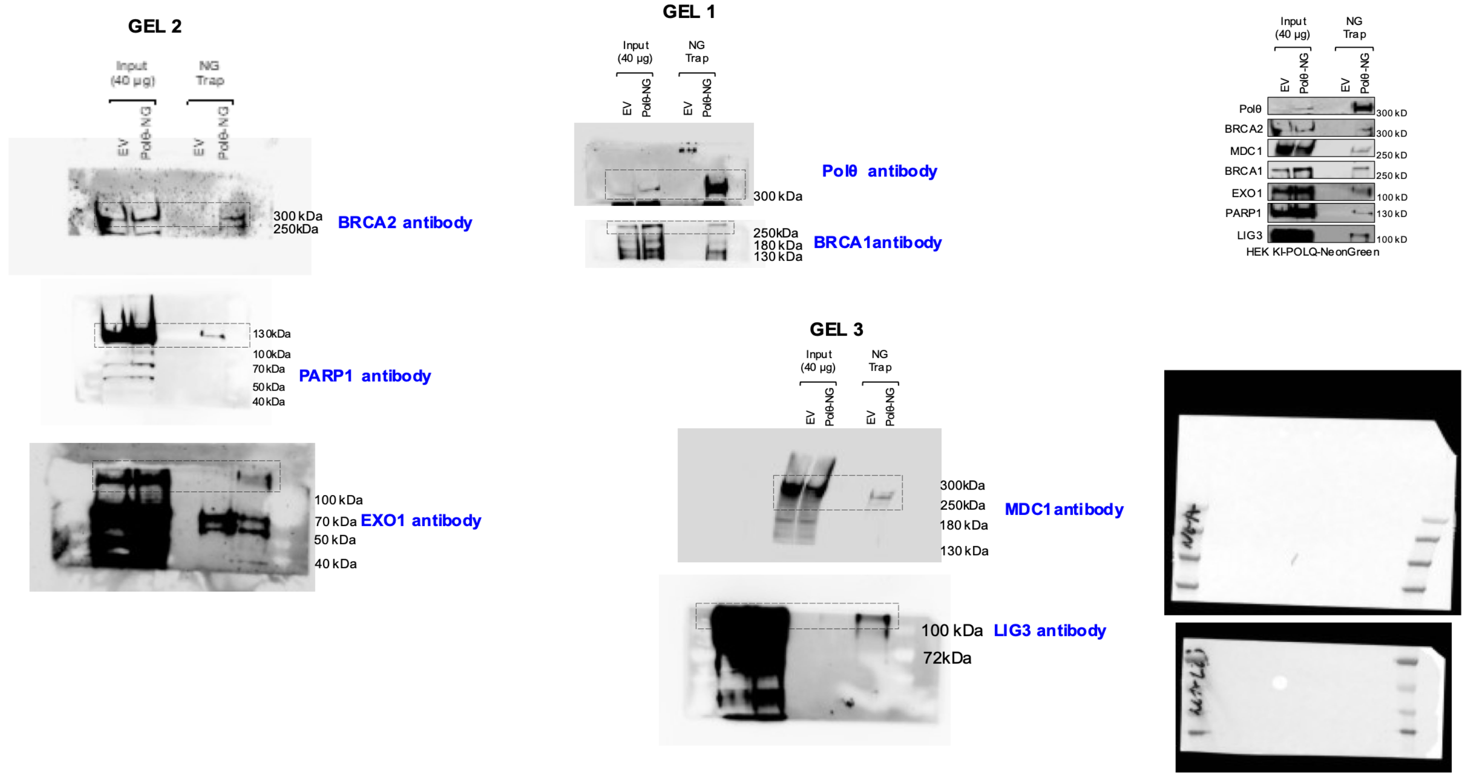

Extended Data Figure 1i. Dotted rectangle correspond to samples included in the manuscript

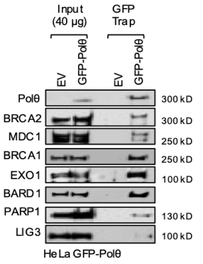

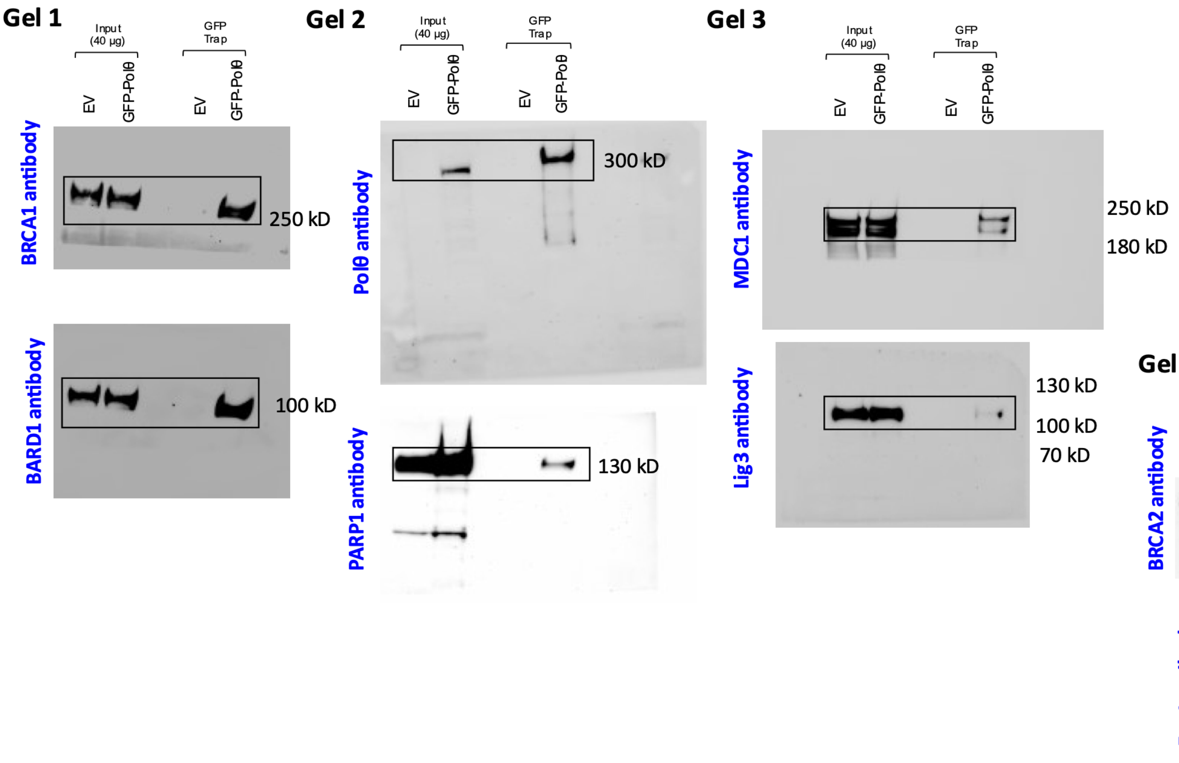

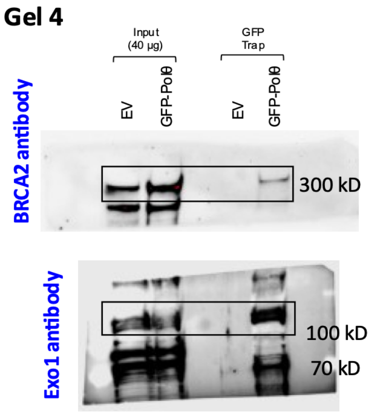

SI Extended Data Figure 4c. Dotted rectangle correspond to samples included in the manuscript

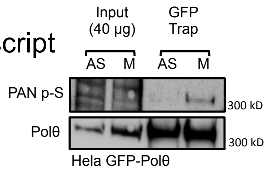

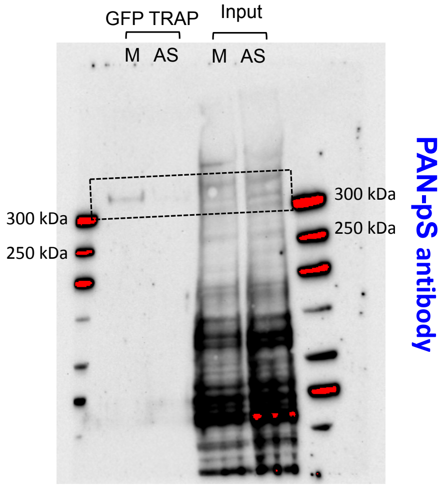

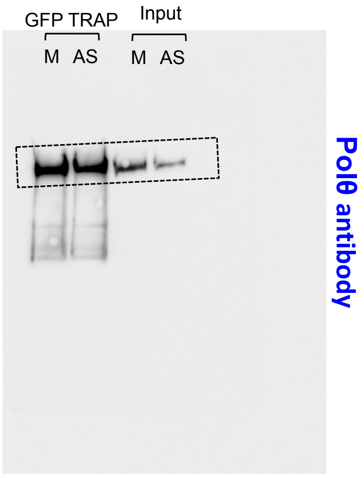

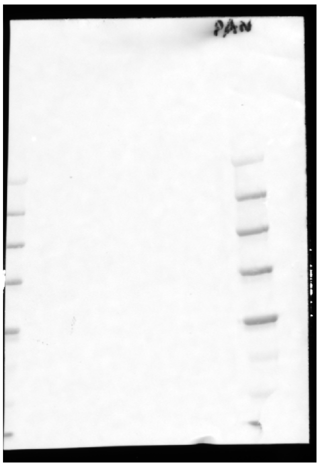

Extended Data Figure 4d. Dotted rectangle correspond to samples included in the manuscript

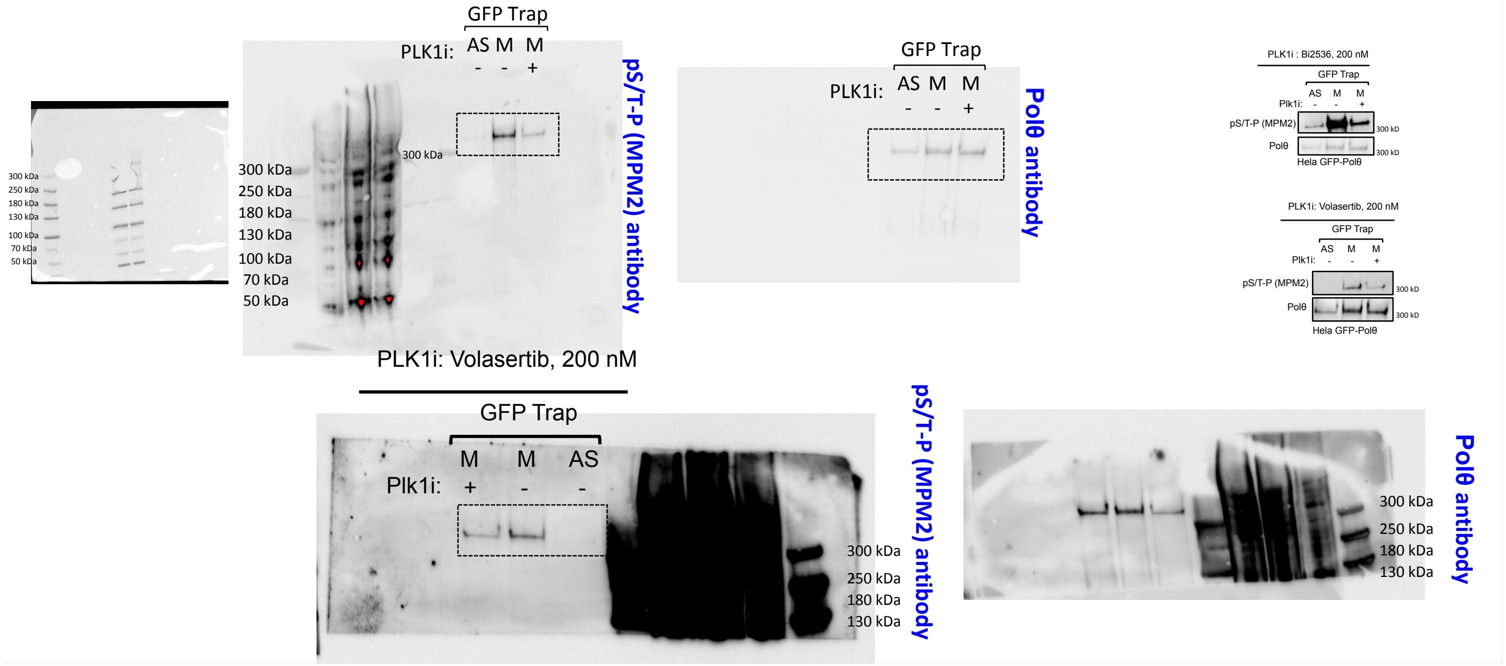

Extended Data Figure 4e. Dotted rectangle correspond to samples included in the manuscript

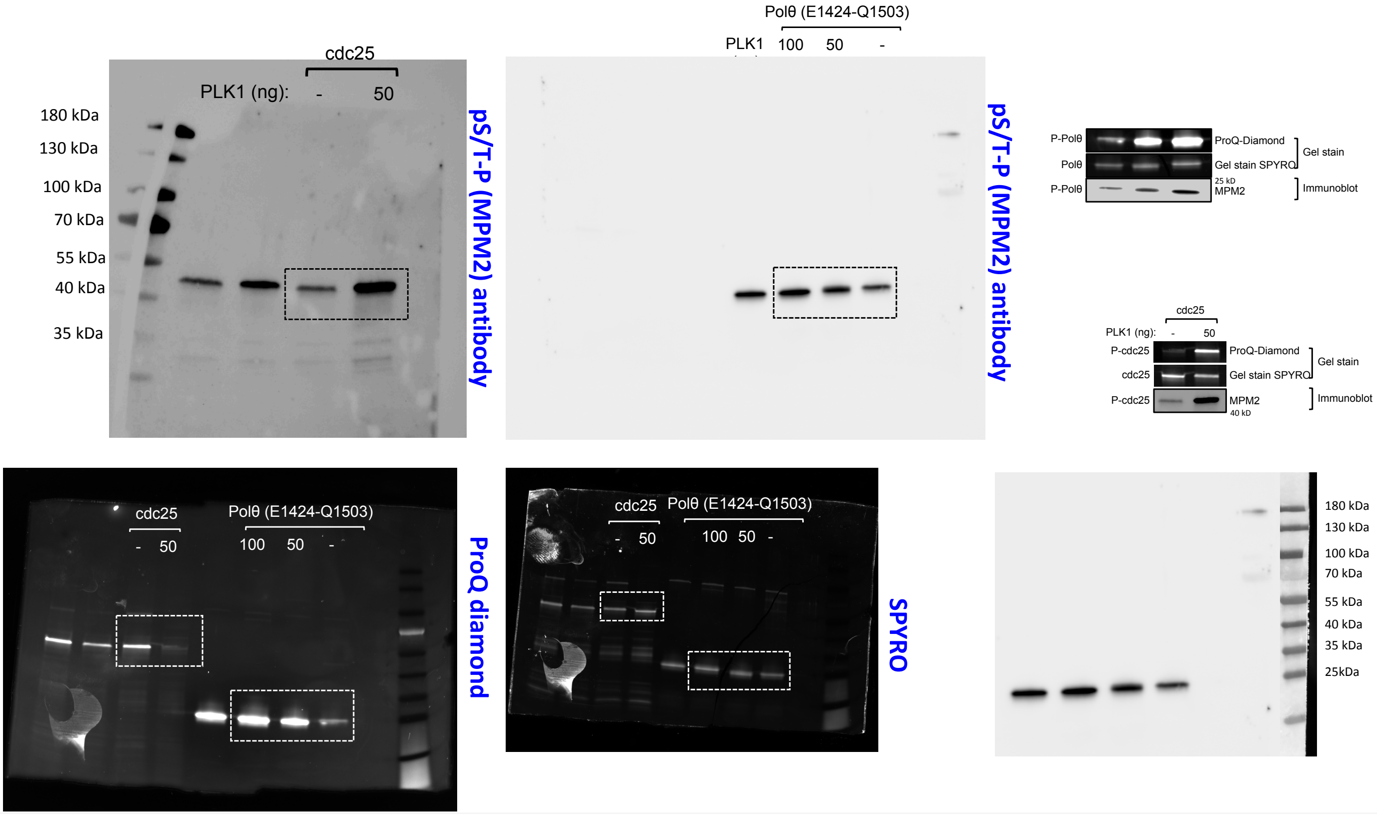

Extended Data Figure 4f. Dotted rectangle correspond to samples included in the manuscript

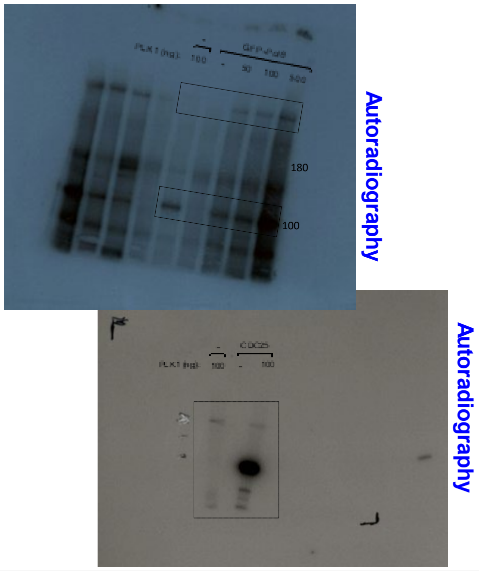

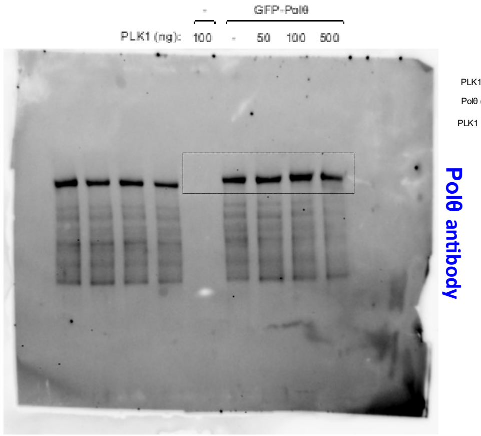

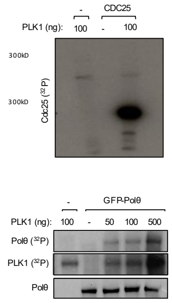

Extended Data Figure 6c. Dotted rectangle correspond to samples included in the manuscript

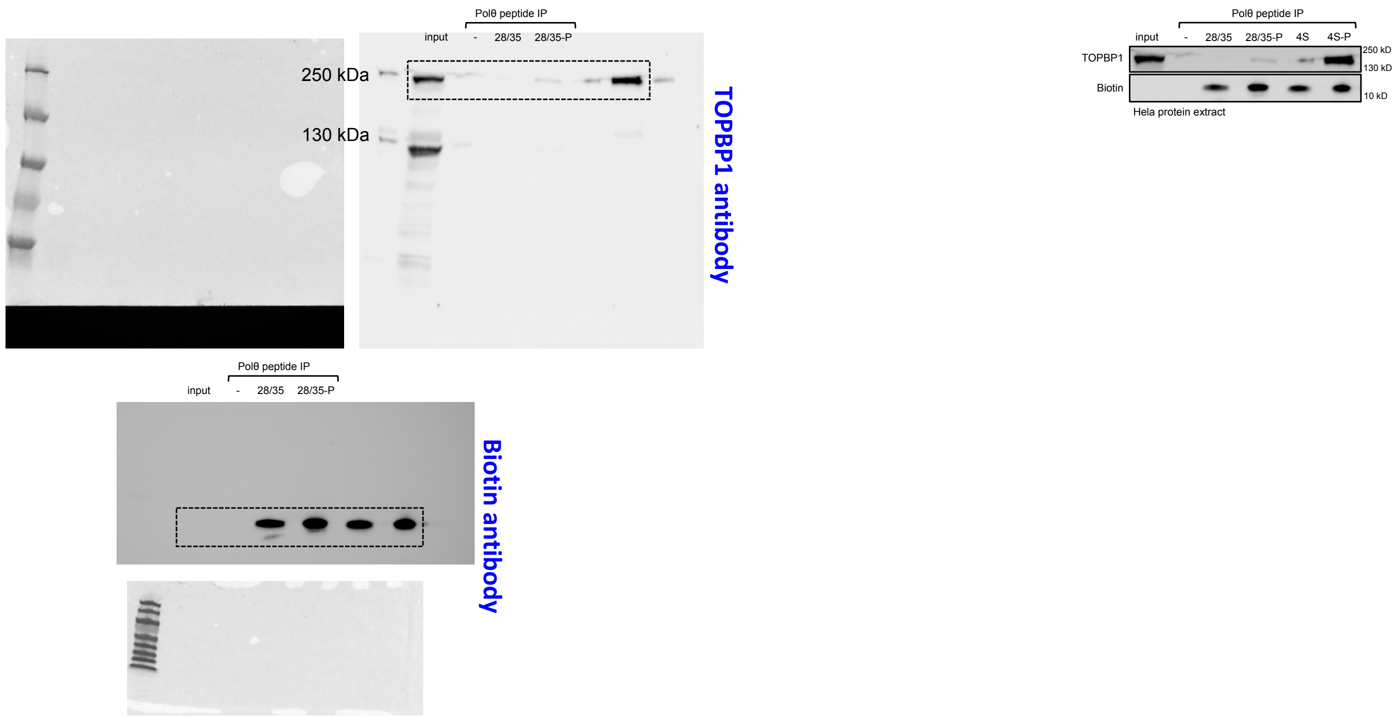

Extended Data Figure 7i. Dotted rectangle correspond to samples included in the manuscript

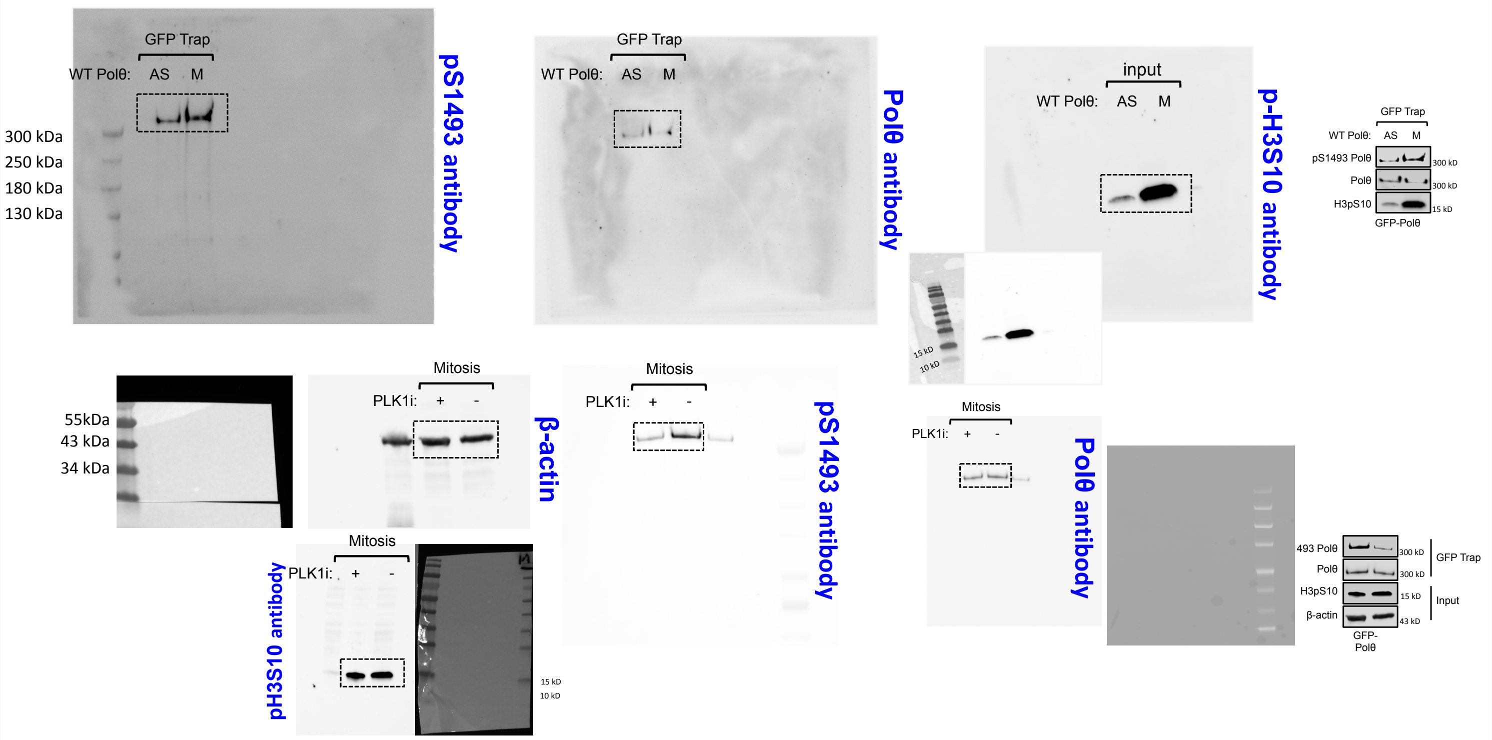

Extended Data Figure 9i. Dotted rectangle correspond to samples included in the manuscript

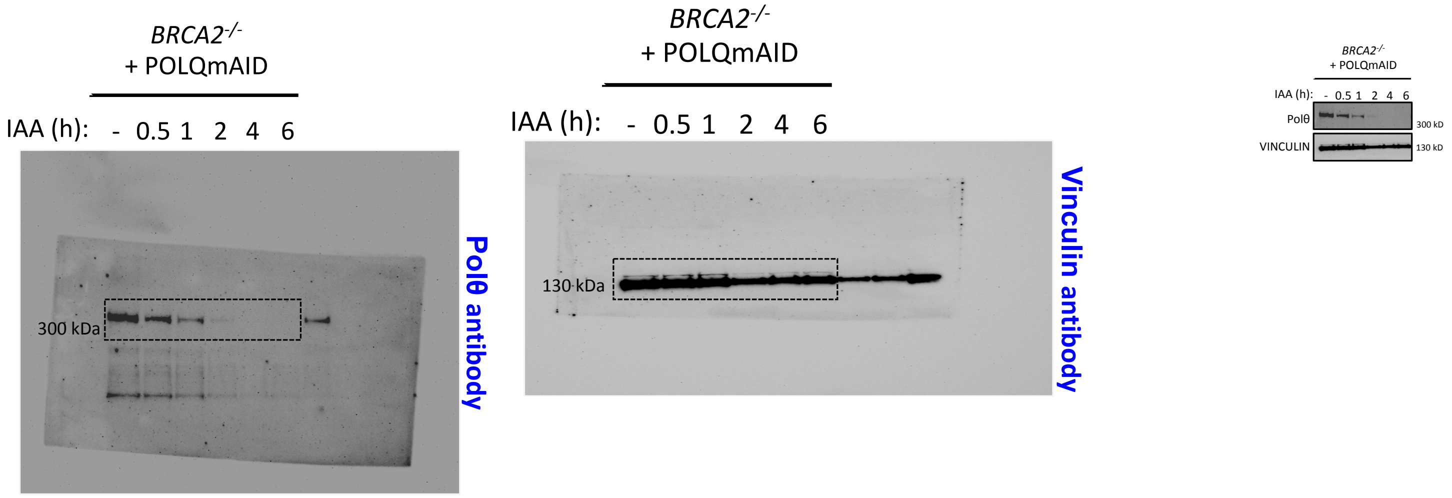

Supplement: Supplementary file 1 — This file contains source data for Figs. 2 and 3 and Extended Data Figs. 1, 4, 6, 7 and 9. In raw gel data, molecular weight markers and the region used in the figure are indicated. [file 41586_2023_6506_MOESM1_ESM.pdf]
