# Supplementary material for: Polθ is phosphorylated by PLK1 to repair double-strand breaks in mitosis
Source: Nature. 2023 Sep 6;621(7978):415–22. doi: 10.1038/s41586-023-06506-6 (PMC10499603; doi:10.1038/s41586-023-06506-6)
Supplement: Supplementary file 6 — List of PLK1 phosphorylation sites identified on Polθ by mass spectrometry. The analysis was performed upon Polθ immunoprecipitation from mitotic HeLa cells were treated with or without PLK1i. [file 41586_2023_6506_MOESM6_ESM.pdf]

Design-based Quantification of Phospho-sites

**Source**		
**Name :**	Phosphos - Noco 500mM/Volacertib 500mM - Norm all	
**Location in myProMS :**	Camille Gelot > Phospho POLQ label free quantifi > Label Free Quantification	
**Design level :**	Experiment	
**Peptide XIC extraction engine :**	MassChroQ (v2.2.21)	
**Labeling :**	Label-free	
**Quantification parameters**		
**Quantification method :**	Protein ratio based on peptide intensity [Simple ratios - Software: myProMS-Quant v3.7]	
**State #1 :**	Volasertib - IP 500mM NaCl: 5 bio. rep. / 5 observations	•X5134FD : XIC extraction 500mM<br>•X5136FD : XIC extraction 500mM<br>•X5138FD : XIC extraction 500mM<br>•X5140FD : XIC extraction 500mM<br>•X5142FD : XIC extraction 500mM
**State #2 :**	Nocodazole - IP 500mM NaCl: 5 bio. rep. / 5 observations	•X5133FD : XIC extraction 500mM<br>•X5135FD : XIC extraction 500mM<br>•X5137FD : XIC extraction 500mM<br>•X5139FD : XIC extraction 500mM<br>•X5141FD : XIC extraction 500mM
**Peptide selection :**	•Proteotypic peptides only.<br>•Missed cleavage allowed.<br>•No modifications allowed.<br>•All charge states.<br>•All sources.<br>•Phospho-sites positions are confirmed if PhosphoRS probability	
**Protein selection :**	All visible proteins	
**Bias correction :**	Median & Scale at peptide level	
**Infinite ratios :**	Not avoided	
**Created :**	Florent Dingli	
**Export settings**		
**Ratios exported :**	•Nocodazole - IP 500mM NaCl/Volacertib - IP 500mM NaCl	
**Fold change ≥**	1.5 (INF=1000)	
**Adj. p-value ≤**	1 (+/-INF allowed)	
**Coefficient of variation ≤**	*No filter*	
**Peptides used for ratio ≥**	1	
**Restrict to proteins in List :**	POLQ > POLQ	

6/22 sites<br>1/1 proteins	Gene &<br>Synonyms	Nocodazole - IP 500mM NaCl/Volasertib - IP 500mM						Total peptides in set	MW (kDa)	Description	Species
		Ratio	Log2 (Ratio)	Adj. p-value	CV %	Dist. pept. used	Pept. used				
O75417-Phospho:S1628.S1639	POLQ,POLH	5,417754	2,4377			1	6	6	289,6	DNA polymerase theta	Homo sapiens
O75417-Phospho:S1289	POLQ,POLH	3,904248	1,965	0,400471	73,9	1	6	8	289,6	DNA polymerase theta	Homo sapiens
O75417-Phospho:T1755	POLQ,POLH	3,242029	1,6969	0,06346	26,9	1	7	8	289,6	DNA polymerase theta	Homo sapiens
O75417-Phospho:S1094*	POLQ,POLH	1,958174	0,9695	0,053612	28,4	1	9	10	289,6	DNA polymerase theta	Homo sapiens
O75417-Phospho:S1594	POLQ,POLH	1,900643	0,9265	0,001049	18,5	2	24	33	289,6	DNA polymerase theta	Homo sapiens
O75417-Phospho:S1436*	POLQ,POLH	1,887173	0,9162	0,115005	38,9	1	10	10	289,6	DNA polymerase theta	Homo sapiens
* Quantified only with re-extracted peptides by Match Between Runs (MBR)											
**Table 4**											
