# Supplementary material for: Polθ is phosphorylated by PLK1 to repair double-strand breaks in mitosis
Source: Nature. 2023 Sep 6;621(7978):415–22. doi: 10.1038/s41586-023-06506-6 (PMC10499603; doi:10.1038/s41586-023-06506-6)
Supplement: Supplementary file 7 — List recording all PLK1 phosphorylation sites on Polθ identified in this study. Sites identified in silico, by NMR and mass spectrometry are indicated. [file 41586_2023_6506_MOESM7_ESM.pdf]

PLK1 phosphorylation sites on Polθ

Amino acid position on Polθ	Predicted to be phosphorylated by PLK1	Phosphorylation motif [D,N,E]-X-[S,T] (elm.eu.org)	Conservation score	Detected by quantitative MS	Detected by NMR	Mutated in 10A-Polθ	Mutated in 4A- and 4D- Polθ
S1289	Yes	LNISRLQ	0	Yes	Not tested	Yes	-
S1321	Yes	FEDSFYL	4	-	Not tested	-	-
S1436	Yes	NEVSVTD	4	Yes (*)	-	-	-
S1444	-		2	-	Yes (**)	-	-
S1482	Yes	PETSLNM	3	N.A	Yes	Yes	Yes
S1486	-		4	N.A	Yes	Yes	Yes
S1488	-		2	N.A	Yes	Yes	Yes
S1493	-		4	N.A	Yes	Yes	Yes
S1551	-		0	N.A	Yes (**)	-	-
S1555	Yes	NDESIIF	3	N.A	Yes	Yes	-
S1563	-		2	-	Yes	Yes	-
T1584	Yes	KNHTVVS	0	-	Yes (**)	-	-
S1594	-		0	Yes	-	-	-
S1628	Yes	QNHSFIW	1	Yes	Yes	Yes	-
S1635	-		3	-	Yes	Yes	-
S1639	-		4	Yes	-	-	-
T1755	-		1	Yes	Not tested	Yes	-

Conservation score
0 = Non conserved
1 = Mammals only
2 = Mammals + Xenops/frogs
3 = Mammals > Birds
4 = Mammals > Fishes

- = No
N.A: Not available
* Quantified only with re-extracted peptides by Match Between Runs (MBR)
** Quantified only after > 5 hours incubation with PLK1. Signal < 80% phosphorylation
