# Supplementary material for: Polθ is phosphorylated by PLK1 to repair double-strand breaks in mitosis
Source: Nature. 2023 Sep 6;621(7978):415–22. doi: 10.1038/s41586-023-06506-6 (PMC10499603; doi:10.1038/s41586-023-06506-6)
Supplement: Supplementary file 8 — List of binding partners to phosphorylated (4S-P) Polθ peptide identified by mass spectrometry. Polθ pepides (4S-P and 4S) were immunoprecipitated in nuclear extracts of HeLa cells. [file 41586_2023_6506_MOESM8_ESM.pdf]

# Design-based Quantification of Proteins

| | | |
|---|---|---|
| **Source** | | |
| **Name :** | F1P/F1 | |
| **Location in myProMS :** | Camille Gelot > 06/12/2022 - Partners of Phospho POLQ - Quantif > Label Free Quantification | |
| **Design level :** | Experiment | |
| **Peptide XIC extraction engine :** | MassChroQ (v2.2.21) | |
| **Labeling :** | Label-free | |
| | | |
| **Quantification parameters** | | |
| **Quantification method :** | Protein ratio based on peptide intensity [Simple ratios - Matching topN peptides - Software: myProMS-Quant v3.8] | |
| **State #1 :** | F1: 5 bio. rep. / 5 observations | •E0981FD : XIC extraction all<br>•E0984FD : XIC extraction all<br>•E0987FD : XIC extraction all<br>•E0990FD : XIC extraction all<br>•E0993FD : XIC extraction all |
| **State #2 :** | F1P: 5 bio. rep. / 5 observations | •E0982FD : XIC extraction all<br>•E0985FD : XIC extraction all<br>•E0988FD : XIC extraction all<br>•E0991FD : XIC extraction all<br>•E0994FD : XIC extraction all |
| **Peptide selection :** | •Proteotypic peptides only.<br>•Missed cleavage allowed.<br>•No modifications allowed.<br>•All charge states.<br>•All sources. | |
| **Protein selection :** | All visible proteins | |
| **Bias correction :** | Median & Scale at peptide level | |
| **Infinite ratios :** | Not avoided | |
| **Created :** | 07/12/2022 09:58:29 by Florent Dingli | |
| | | |
| **Export settings** | | |
| **Ratios exported :** | •F1P/F1 | |
| **Fold change ≥** | 2 (INF=1000) | |
| **Adj. p-value ≤** | *User selection* | |
| **Coefficient of variation ≤** | *User selection* | |
| **Peptides used for ratio ≥** | 3 | |
| **Sort by :** | Peptides used | |
| **Restrict to proteins in List :** | Dam > Up in F1P & F1 /beads >3 5PD 5rep | |

| 25/72 proteins | Gene & Synonyms | F1P/F1 | | | | | | Total peptides in set | MW (kDa) | Description | Species |
|---|---|---|---|---|---|---|---|---|---|---|---|
| | | Ratio | Log2(Ratio) | Adj. p-value | CV % | Dist. pept. used | Pept. used | | | | |
| P53621 | COPA | 2,310036 | 1,207915 | 5,94E-59 | 5,457941 | 67 | 809 | 999 | 138,3 | Coatomer subunit alpha | Homo sapiens |
| P46940 | IQGAP1,KIAA0051 | 8,673016 | 3,116534 | 5,1E-184 | 2,339064 | 75 | 722 | 1042 | 189,3 | Ras GTPase-activating-like protein IQGAP1 | Homo sapiens |
| P12270 | TPR | 7,377798 | 2,88319 | 8E-120 | 3,122108 | 66 | 580 | 1107 | 267,3 | Nucleoprotein TPR | Homo sapiens |
| O75165 | DNAJC13,KIAA0678,RME8 | 3,662649 | 1,872887 | 1,76E-52 | 5,286823 | 51 | 404 | 586 | 254,4 | DnaJ homolog subfamily C member 13 | Homo sapiens |
| Q14789 | GOLGB1 | 6,535571 | 2,708313 | 9,91E-93 | 3,317698 | 46 | 380 | 806 | 376,0 | Golgin subfamily B member 1 | Homo sapiens |
| Q9Y4P3 | TBL2,WBSCR13,UNQ563/PRO1125 | 2,005983 | 1,004309 | 4,77E-16 | 10,86294 | 27 | 302 | 404 | 49,8 | Transducin beta-like protein 2 | Homo sapiens |
| Q9Y305 | ACOT9,CGI-16 | 2,397907 | 1,261775 | 3,87E-17 | 10,13246 | 23 | 223 | 276 | 49,9 | Acyl-coenzyme A thioesterase 9, mitochondrial | Homo sapiens |
| O15078 | CEP290,BBS14,KIAA0373,NPHP6 | 2,573007 | 1,363455 | 1,92E-18 | 9,507075 | 34 | 203 | 372 | 290,4 | Centrosomal protein of 290 kDa | Homo sapiens |
| P83111 | LACTB,MRPL56,UNQ843/PRO1781 | 4,509831 | 2,173073 | 8,16E-44 | 5,014934 | 21 | 198 | 257 | 60,7 | Serine beta-lactamase-like protein LACTB, mitochondrial | Homo sapiens |
| Q9P1Y5 | CAMSAP3,KIAA1543 | 3,322523 | 1,732279 | 2,44E-21 | 8,607837 | 25 | 196 | 277 | 134,8 | Calmodulin-regulated spectrin-associated protein 3 | Homo sapiens |
| Q13769 | THOC5,C22orf19,KIAA0983 | 7,783691 | 2,960455 | 2,46E-50 | 4,305671 | 23 | 187 | 316 | 78,5 | THO complex subunit 5 homolog | Homo sapiens |
| Q9P0L0 | VAPA,VAP33 | 3,44386 | 1,784027 | 1,1E-20 | 8,738599 | 10 | 183 | 208 | 27,9 | Vesicle-associated membrane protein-associated protein A | Homo sapiens |
| Q92547 | TOPBP1,KIAA0259 | 2,596899 | 1,37679 | 1,92E-18 | 9,335861 | 21 | 175 | 313 | 170,7 | DNA topoisomerase 2-binding protein 1 | Homo sapiens |
| Q96FV9 | THOC1,HPR1 | 4,356188 | 2,123066 | 4,96E-27 | 6,958328 | 15 | 159 | 272 | 75,7 | THO complex subunit 1 | Homo sapiens |
| Q13393 | PLD1 | 4,57721 | 2,194468 | 1,66E-22 | 7,865281 | 22 | 152 | 294 | 124,2 | Phospholipase D1 | Homo sapiens |
| O14579 | COPE | 2,628099 | 1,39402 | 1,82E-16 | 9,696928 | 10 | 133 | 165 | 34,5 | Coatomer subunit epsilon | Homo sapiens |
| Q9UBH6 | XPR1,SYG1,XR | 2,369075 | 1,244324 | 4,76E-18 | 8,380219 | 7 | 95 | 98 | 81,5 | Xenotropic and polytropic retrovirus receptor 1 | Homo sapiens |
| Q13190 | STX5,STX5A | 2,080326 | 1,05681 | 1,54E-09 | 13,35044 | 8 | 92 | 121 | 39,7 | Syntaxin-5 | Homo sapiens |
| Q9Y6X9 | MORC2,KIAA0852,ZCWCC1 | 3,695243 | 1,885669 | 1,66E-13 | 10,2495 | 13 | 90 | 129 | 117,8 | ATPase MORC2 | Homo sapiens |
| Q86W42 | THOC6,WDR58,PSEC0006 | 8,64146 | 3,111275 | 3,31E-19 | 7,04894 | 10 | 74 | 114 | 37,5 | THO complex subunit 6 homolog | Homo sapiens |
| O75528 | TADA3,ADA3,TADA3L | 2,407508 | 1,267541 | 5,62E-06 | 17,82711 | 8 | 68 | 82 | 48,9 | Transcriptional adapter 3 | Homo sapiens |
| Q96J01 | THOC3 | 2,258921 | 1,175634 | 7,36E-05 | 20,40859 | 6 | 60 | 119 | 38,8 | THO complex subunit 3 | Homo sapiens |
| Q4VC44 | FLYWCH1,KIAA1552 | 8,956446 | 3,162926 | 1,36E-27 | 4,053471 | 7 | 59 | 96 | 80,1 | FLYWCH-type zinc finger-containing protein 1 | Homo sapiens |
| P57772 | EEFSEC,SELB | 7,530191 | 2,912686 | 5,16E-18 | 5,846612 | 5 | 46 | 112 | 65,3 | Selenocysteine-specific elongation factor | Homo sapiens |
| Q9Y485 | DMXL1,XL1 | 6,878255 | 2,782043 | 3,86E-08 | 8,526515 | 3 | 21 | 182 | 337,8 | DmX-like protein 1 | Homo sapiens |
